# Enhanced unbiased sampling of protein dynamics using evolutionary coupling information

**DOI:** 10.1038/s41598-017-12874-7

**Published:** 2017-10-05

**Authors:** Zahra Shamsi, Alexander S. Moffett, Diwakar Shukla

**Affiliations:** 1 Department of Chemical and Biomolecular Engineering, University of Illinois, Urbana, IL 61801 USA; 2 Center for Biophysics and Quantitative Biology, University of Illinois, Urbana, IL 61801 USA; 3 Department of Plant Biology, University of Illinois, Urbana, IL 61801 USA; 4 National Center for Supercomputing Applications, University of Illinois, Urbana, IL 61801 USA

## Abstract

One of the major challenges in atomistic simulations of proteins is efficient sampling of pathways associated with rare conformational transitions. Recent developments in statistical methods for computation of direct evolutionary couplings between amino acids within and across polypeptide chains have allowed for inference of native residue contacts, informing accurate prediction of protein folds and multimeric structures. In this study, we assess the use of distances between evolutionarily coupled residues as natural choices for reaction coordinates which can be incorporated into Markov state model-based adaptive sampling schemes and potentially used to predict not only functional conformations but also pathways of conformational change, protein folding, and protein-protein association. We demonstrate the utility of evolutionary couplings in sampling and predicting activation pathways of the *β*_2_-adrenergic receptor (*β*_2_-AR), folding of the FiP35 WW domain, and dimerization of the *E. coli* molybdopterin synthase subunits. We find that the time required for *β*_2_-AR activation and folding of the WW domain are greatly diminished using evolutionary couplings-guided adaptive sampling. Additionally, we were able to identify putative molybdopterin synthase association pathways and near-crystal structure complexes from protein-protein association simulations.

## Introduction

## Background

Molecular dynamics (MD) simulation has rapidly advanced into an invaluable tool for understanding the structure-function relationship in biological molecules and providing specific, testable predictions in molecular biology^1–6^. However, a key limitation of MD simulation is the difficulty of efficiently sampling conformational ensembles where dynamics take place over computationally vast time-scales^7,8^. Countless innovations such as steered MD^9^, accelerated MD^10^, and replica exchange MD^11^ improve the efficiency of sampling but either require subjective choices of reaction coordinates or sacrifice kinetic information for accurate thermodynamics. Regardless of their limitations, these methods and others have achieved great success and have allowed for analysis of protein structure and dynamics in unprecedented detail^12^.

At the same time, thorough experimental investigation is often a prerequisite for the use of MD in order to determine an accurate initial set of coordinates for simulation, although the ever-growing database of protein structures^13^ coupled with homology modeling methods have weakened this constraint^14–17^. Computational biophysicists must further rely on experimental data in order to identify biologically relevant structures from conformational ensembles created through simulation, a task which would otherwise require often prohibitively expensive simulations with large numbers of atoms and periods of time or methods able to capture chemical processes, such as combined quantum and classical simulations (QM/MM)^18^. In order to move beyond using biomolecular simulation to explain experiments *post hoc* and better capture the predictive power of molecular simulation, methods allowing biologically relevant conformational states to be identified by computational means must be developed.

Recently developed bioinformatic methods have had success in elucidating native contacts within single-chain proteins and between subunits of complexes from sequence information alone^19–24^. Computationally tractable methods of estimating global sequence probabilities using mean field and pseudolikelihood maximization approximations have been shown to be capable of extracting evolutionarily coupled pairs of residues from multiple sequence alignments of homologous proteins^19,25,26^. With the assumption that evolutionarily coupled residues likely form contacts critical for function leading to a strong selective force against proteins where one residue is mutated to an amino acid that changes the nature of the interaction in a coupled pair, one can infer functional contacts between residues of proteins allowing for the successful prediction of protein folds^19,20^ and dimer structures^21–23^ using energetic distance restraints in concert with docking methods. While this assumption that covariance of residues indicates coevolution raises some questions^27^, the successes of structure prediction methods founded upon this assumption are undeniable, and it has been shown that the assumption of spatial proximity of coupled residues is a well founded one^28^. Overall, the ability to use evolutionary information encoded in the sequences of protein families to recognize functionally important inter-residue distances allows for the unique ability to predict structures with little *a priori* structural information and is therefore an enticing tool for computational structural biologists.

These methods of identifying evolutionary couplings have led to a number of papers feeding evolutionary coupling information into molecular simulations in the form of energetic distance restraints in order to predict native structures and create conformational ensembles consistent with the inferred contacts between coupled residues in an active conformation^24,29–32^. While these methods have great value in their ability to predict new quasi-static structures or restrained ensembles likely to represent conformational states of biological importance, they all either operate under the assumption that evolutionarily coupled residues will more or less be in contact with one another throughout the lifetime of the protein or use energetic biasing and coarse-grained models meaning that dynamics between structures will have little relevance to the actual physical behavior of a protein. However, past structural and computational studies have indicated that large degrees of conformational heterogeneity exist in protein ensembles^33–36^. This indicates that actual proteins in their cellular environment will likely not have all of these coupled residues in contact with one another all the time, particularly for signaling proteins such as kinases and G protein-coupled receptors (GPCRs) which must respond selectively to signals^37,38^. With this in consideration, we aim in this paper to evaluate the use of distances between evolutionarily coupled residues as bioinformatics-derived reaction coordinates describing pathways between biologically incompetent and competent structures.

### Evolutionary couplings as reaction coordinates

In order to exploit the information provided by evolutionary couplings for use in atomistic simulations of proteins, we have developed evolutionary couplings-guided adaptive sampling (ECAS). Since statistical coupling between a pair of residues over the process of molecular evolution is believed to be caused by selection for a *functional* physical interaction, we can assume that residue pairs with strong coupling scores form interactions necessary for some unknown function of the protein. We take the pairs of residues with strong coupling scores and use the distances between them to guide sampling towards a functional conformation, which should be characterized by small distances between coupled residue pairs. Conversely, non-functional conformations will likely have large distances between coupled residue pairs.

We use adaptive sampling, where no biasing terms are added to the system Hamiltonian but directional information can be used by iteratively running unbiased simulations and choosing conformations which satisfy some condition, in this case the smallest or largest distances between coupled residue pairs, to start the next round of sampling from. In this way, when the process of interest is conformational changes of folded proteins, we avoid pulling apart residues which may have strong evolutionary coupling scores but are important for folding and remain in contact for all biologically relevant conformations. This is because ECAS exploits equilibrium fluctuations in the distances between residue pairs, which should be small for contacts keeping the overall fold of the protein. ECAS is related to the FAST algorithm^39^ with the undirected component of the reward function set to zero, and using distances between evolutionarily coupled residues. Rather than energetically biasing our systems, our method intentionally introduces sampling bias, which can effectively be removed by constructing a Markov state model on the protein dynamics (see Supplementary Information for further discussion of bias in adaptive sampling)^40^.

For signaling proteins known to possess more than one functional conformational state (in the simplest case, “on” and “off” states) it is unclear which coupled residue pair contacts will form in each of these functional states. For our ECAS scheme, we take the sum of all distances between each pair of coupled residues for each frame and subtract the same value calculated for a reference structure representing a known functional state, which yields a number (the change in the sum of evolutionary couplings pair distances, or ΔSEC) used as a score for selecting starting structures in adaptive sampling. Structures generated from unbiased MD simulation with high ΔSEC values are by one measure the most different from the reference structure in the evolutionarily coupled degrees of freedom. Therefore adaptive sampling using ΔSEC does not require knowledge of the functional role of contacts between coupling residue pairs in a particular system as the scheme is specifically designed to seek out overall change in distances between coupled residues without regard for the direction of change for specific distances.

To first demonstrate the general utility of directional knowledge in enhancing sampling we perform directionally-guided adaptive sampling on a rugged two-dimensional potential. We assume that there is a known “evolutionary coupling” in this system, which is the distance to a deep potential energy well located at the origin of the system, and we show the intuitive improvement of progress towards the “active” state when trajectories are iteratively clustered and starting points for the subsequent rounds are chosen from clusters with minimal distances to the “active” state. Next, to show how evolutionary couplings can be used to both predict biologically competent structures and to reduce simulation time needed to reach these structures of interest, we perform evolutionary couplings-guided adaptive sampling to sample Markov state models (MSMs) built from previously published extensive MD simulation of the *β*_2_ adrenergic receptor (*β*_2_-AR)^38^ and of the FiP35 WW domain^41^. Finally, we use distances between evolutionarily coupled residues in a modified scheme described below to qualitatively characterize the association pathways and dominant dimeric structures formed by the subunits of the *E. coli* molybdopterin synthase (MoaD and MoaE), at the same time allowing for a reasonable blind prediction of the native structure corresponding to the structure with the lowest sum of distances between coupled residue pairs.

## Methods

### Calculation of evolutionary couplings

Evolutionary couplings are correlations in amino acid occurrence due to direct interaction between residues, and can therefore be used to infer contacts in native protein structures. Simple calculation of covariance between residues through groups of homologous sequences does not differentiate between correlations due to direct physical interactions and indirect interactions through intermediating residues. In order to infer direct couplings between residues from multiple sequence alignments (MSA) of homologous protein sequences, a more sophisticated statistical method is required. We used the pseudolikelihood method on the EVCouplings web server^42^ with default settings to obtain evolutionary couplings, except where noted. See Ekeberg *et al*.^26^ for a detailed description of the method.

### Markov state models

MSMs are kinetic models which represent protein dynamics as a Markov chain on discretizations of conformational space achieved using clustering algorithms on sets of MD trajectories^43^. Transitions between states in MD trajectories are counted, and from these counts a maximum likelihood transition probability matrix is estimated^44^. The behavior over time of any given initial probability mass function over the states, the row vector ${\bf{p}}({t}_{0})$, can be given by:

1 $${\bf{p}}({t}_{0}+k\tau )={\bf{p}}({t}_{0}){\bf{T}}{(\tau )}^{k}$$

where ${\bf{T}}(\tau )$ is the transition probability matrix, a right stochastic matrix constructed at a lag time of $\tau$ (an element of ${\bf{T}}(\tau )$ is represented as ${p}_{ij}$). MSMs allow for accurate approximation of protein dynamical process timescales far longer than any individual trajectory used in MSM construction^45^, and importantly for adaptive sampling, allow for estimation of the equilibrium populations of states from trajectories sampled from non-equilibrium distributions^40^. All MSM analysis in this study was conducted using the MSMBuilder 3 Python package^46^.

### Adaptive sampling

The adaptive sampling method involves iteratively running short simulations in parallel, clustering on a relevant structural metric, and seeding new simulations from clusters based on some criterion. Adaptive sampling has been shown to sample configurational space more efficiently than the simulated tempering method for simulation of an RNA hairpin^40^. Several methods for selecting clusters to choose seeding structures have been explored, including randomly picking a fixed number of structures from each cluster^40^, picking structures from states which have the greatest contributions to the statistical error of MSMs built after each clustering step^47^, and picking structures from states with the lowest raw counts^48^. The specific methods we have used for each system are detailed in their respective methods sections.

### Kinetic Monte Carlo on Markov state models

Kinetic Monte Carlo is a method for sampling from a kinetic model which can be used to create trajectories of state-to-state dynamics. If the initial state is chosen to be $i$, a transition to any state $j$ in the set of all states in the MSM occurs with probability ${p}_{ij}$ from the reversible maximum-likelihood transition matrix. This is practically implemented by generating a pseudo-random number between zero and one and taking a cumulative sum of ${p}_{ij}$ values over $j$ (${S}_{n}={\sum }_{j\mathrm{=1}}^{n}{p}_{ij}$); then if the pseudo-random number lies between ${S}_{n}$ and ${S}_{n+1}$, there will be a transition to state *j* = *n* + 1. This state is added to the trajectory and the process is repeated for the desired number of steps.

### Transition path theory

Transition path theory (TPT) allows for characterization of reactive probabilities and fluxes in order to determine the likelihood of, in this case, two proteins binding given that they are in a certain state to start, along with pathways in the MSM between two states with the highest probability flux. We used the MSMExplorer implementation of TPT, using Dijkstra’s algorithm^49^ in order to identify top pathways from the net flux matrix. For a detailed overview of TPT, we refer the reader to a recent review by Vanden-Eijnden *et al.* ^50^.

### Simulation of a two-dimensional Brownian particle

We used the Euler-Maruyama approximation^51^ to integrate the equations of motion of a two-dimensional Brownian particle on an external potential (see Supplementary Information for details). Three sampling protocols were compared: long serial simulations, random adaptive sampling, and evolutionary couplings-guided adaptive sampling. Serial simulations involved integrating a 10 independent trajectories in parallel for 300,000 steps, while adaptive sampling involved running 10 trajectories for 10,000 steps each and then clustering into 100 states and choosing 10 new starting points for another round of sampling. The adaptive sampling process was then repeated for a total of 30 rounds of sampling, yielding equivalent amounts of data for all three strategies. Random adaptive sampling means that the 10 states are chosen randomly (without replacement), and a single point is chosen randomly from each selected state, while evolutionary couplings-guided adaptive sampling is identical except that the 10 states are chosen based on their average distance (over all points in a particular cluster) to the “active” position, where the 10 lowest average distance clusters are selected.

### Kinetic Monte Carlo sampling on a *β*_2_-AR Markov state model

We used previously published all-atom molecular dynamics simulations of *β*_2_-AR^38^, initiated from the active state crystal structure (PDB ID: 3P0G^52^) and performed using the CHARMM27 (Chemistry at Harvard Molecular Mechanics) force field^53^ on the Anton supercomputer^54^. All simulations were performed in NPT (310 K, 1 bar) conditions with the explicit presence of a lipid membrane and water^38^. Twenty-five simulations totaling 166 *μs* for agonist-bound *β*_2_-AR with the Nb80 nanobody removed were initiated from the active structure with protonated D130^3.49^ (Ballesteros-Weinstein numbering)^55^. The simulations sampled the active, intermediate, and inactive states of *β*_2_-AR.

Trajectories from *β*_2_-AR simulations were featurized using *ϕ*, *ψ*, and ${\chi }_{1}$ protein dihedral angles. In order to reduce the dimensionality of the feature space, time-lagged independent components analysis (tICA)^56,57^ was performed and trajectories were projected onto the ten slowest tICs for clustering. An MSM with 1000 microstates and a lag time (*τ*) of 50 ns, selected based on convergence of implied timescales (Supplementary Fig. S1), was constructed. All later Monte Carlo simulations of *β*_2_-AR in this study were performed on this reference MSM.

The same three simulation strategies employed in the Brownian particle simulations were used in *β*_2_-AR simulations, and the time required to reach the active state was calculated for each sampling method multiple times with different seeding parameters. In the traditional long simulations, varying numbers of parallel simulations starting from the inactive state were run for varying amounts of time. The procedure was performed on a total of 3300 sets of simulations of different lengths and numbers of trajectories, where the lengths (*S*) varied from 1 *τ* to 5000 *τ* and numbers of parallel trajectories (*N*) varied from 1 to 1000. In random sampling, *N* parallel trajectories were started from the inactive state in the reference MSM and run for a time of *S* each. From the resulting trajectories on the reference MSM, *N* microstates were picked randomly to start another set of *S* ns long simulations, beginning the second adaptive round. This procedure was continued for *R* adaptive rounds. Again, 3300 sets of simulations were performed, where *N* was kept constant at 10, *S* was varied from 1 *τ* to 5000 *τ* and *R* was varied from 0 to 100. Lastly, evolutionary couplings-guided adaptive sampling was performed in an identical manner to random adaptive sampling except that after every round of simulation, the *N* microstates with the maximal values of ΔSEC for the 800 residue pairs with the highest coupling scores (over a chosen cutoff score of 0.012, where the number of couplings was rounded) (Supplementary Figs S2 and S3) with respect to the inactive crystal structure, were chosen for the next round of simulation. The ΔSEC value for each microstate was calculated by taking the average ΔSEC value over 50 randomly chosen member structures.

### Kinetic Monte Carlo sampling on a FiP35 WW domain Markov state model

In order to test the performance of the proposed method in sampling the folding of a protein, we used previously reported simulations of the folding of the FiP35 WW domain^41^, where one millisecond of all-atom molecular dynamics simulations were performed on the Anton supercomputer using the Amber ff99SB-ILDN force field and the TIP3P water model^58,59^.

We used the distribution of reciprocal interatomic distances^60^ as a featurization metric to cluster the MD trajectories into 2000 microstates and build an MSM in the same manner as *β*_2_-AR. An MSM with a lag time (*τ*) of 120 ns, selected based on convergence of implied timescales (Supplementary Fig. S4), was constructed, faithfully reproducing the raw data. All later Monte Carlo simulations of FiP35 WW domain in this study were performed on this reference MSM. The performance of the three simulation methods tested for the *β*_2_-AR MSM (see Supplementary Fig. S5 for evolutionary coupling differences between folded and unfolded states) was evaluated by determining how much time it took to observe the complete folding process starting from an arbitrary unfolded state. The three different approaches were used each in 3000 sets of simulations of different lengths and numbers of trajectories where the total time to reach the folded state was calculated for each, in the same manner as *β*_2_-AR, except that the lengths of trajectories ranged from 1 *τ* to 30 *τ* and 70 evolutionarily coupled residue pairs (the number of pairs with coupling scores over an arbitrarily chosen cutoff score of 0.012, where the number of couplings was rounded) were used.

### Simulations of MoaD-MoaE dimerization

Simulations of the dimerization of the two *E. coli* molybdopterin synthase subunits, MoaD and MoaE, were performed using the crystal structure of the individual proteins (PDB ID: 1FM0^61^) in the AMBER14^62^ molecular dynamics package using the AMBER14SB forcefield^63^, and set up using AMBERTools 14^64^. All simulations were performed in the Generalized Born Neck 2 implicit solvent model^65^ using Langevin dynamics with a 2 fs timestep and a collision frequency of 2 ps^−1^ maintaining a temperature of 300 K. Simulations were performed on the Blue Waters petascale computing facility at National Center for Supercomputing Applications. The two monomers were separated so that the distance between their centers of mass was approximately 50 Å. The initial structure was subjected to energy minimization for 10,000 steps, and equilibrated for 1 ns. A single simulation was started from the equilibrated structure and run for 100 ns and the resulting trajectory was clustered into 25 clusters on the distances between the five residue pairs with the highest EV complex scores^22^ using the K-means implementation in the Scikit-learn Python module^66^. Nearest neighbors of cluster means were chosen using the Scikit-learn K-neighbors implementation and were used to start a new round of sampling, consisting of 25 simulations running in parallel for 200 ns each. The resulting 5 *μ*s of sampling was clustered in same manner as before into 200 clusters, and the cluster centers from the 50 lowest populated states were selected as starting points for the next round. The ten subsequent sampling rounds consisted of 50 simulations run in parallel for 100 ns, a total of 5 *μ*s per round, and were clustered in the same 200 cluster, 50 lowest populated state manner to generate starting structures for the next round. In order to reduce size of datasets for analysis, all trajectories were subsampled by every 100^*th*^ frame so that each frame in the subsampled trajectories represented 200 ps of simulation.

Trajectories from simulation of MoaD-MoaE dimerization were featurized by their centers of mass using the MDTraj Python package^67^. For each frame, the MoaD center of mass vector was subtracted from the MoaE center of mass vector and a set of normalized basis vectors were defined from MoaD atom coordinates (see Supplementary Information for details). MoaD was chosen to define the coordinate system due to the relatively narrow distribution of its RMSD with respect to its crystal structure (Supplementary Fig. S6). The trajectories of the MoaE center of mass were then projected onto the MoaD basis sets for clustering. While using the relative translational orientations of the two proteins for construction of an MSM obscures both the rotational and internal conformational degrees of freedom, the utilized features should be sufficient to identify metastable states in terms of relative orientation. Furthermore, ignoring slowly evolving internal degrees of freedom prevents creation of a separation of timescales, which can complicate MSM construction^68^.

Structures on the MoaD basis set were clustered with the K-means algorithm into 500 states and a maximum likelihood transition probability matrix was estimated based on transition counts between states using a lag time (*τ*) of 40 ns, selected based on convergence of implied timescales (Supplementary Fig. S7). Top flux pathways from the state with average Cartesian coordinates closest to the starting structure to the state closest to the crystal structure was produced using the MSMExplorer application^69^.

## Results

### Informed reaction coordinates dramatically enhance conformational sampling efficiency

**Figure 1:**
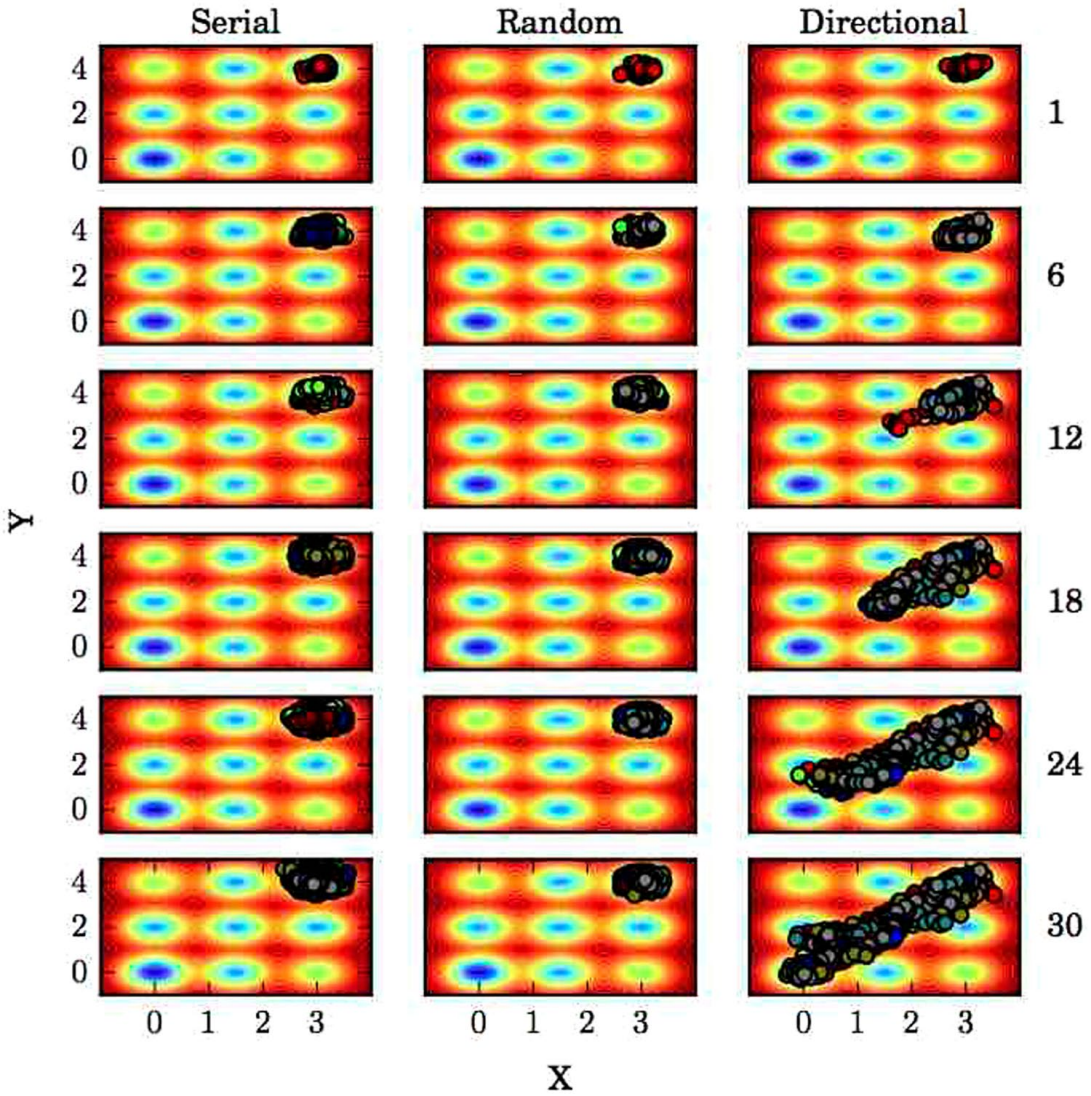
Guided sampling on a potential energy surface. Brownian dynamics simulations using the traditional serial, long simulation method, random adaptive sampling, and directionally-guided adaptive sampling. Data points from sampling rounds prior to and including the indicated round to the right of the plots are displayed, with different colors for different sampling rounds.

As an illustration of the utility of directional knowledge for enhancing adaptive sampling, we simulated a Brownian particle in two dimensions on a rugged potential energy surface using long serial simulations, random adaptive sampling, and directionally-guided adaptive sampling with the intent of sampling a pathway between the two deepest energy minima, from the top right-hand corner to the origin (Fig. 1). With serial trajectories, the Brownian particle has a relatively low probability of leaving the starting energy minimum well, and in the particular example provided in Fig. 1 the particle was unable to do so. This can be understood conceptually with reference to transition state theory, where the energy barrier between metastable wells, analogous to the activation energy of a chemical reaction, limits the rate of transition. However, if sampling is done in rounds where new trajectories are initiated from positions closer to the “transition state” saddle point, a trajectory resulting in the particle crossing this barrier and into the next minimum well is more likely than for trajectories initiated at more distant points within the initial minimum well. With no directional information available, one strategy is to randomly choose clusters to seed trajectories from, which can be of use^40^, but fails to improve sampling in the particular example shown in Fig. 1. If directional information is available, that is, if it there is some structural information known about the target state that differs from the starting state, we can exploit that information by choosing clusters from each round of sampling that have a minimal average (over structures contained in a particular cluster) distance in the known distinguishing metrics to the target state. This drives the system towards the target state without any kind of energetic bias (Fig. 1).

### Efficient sampling of *β*_2_-AR activation pathways

**Figure 2:**
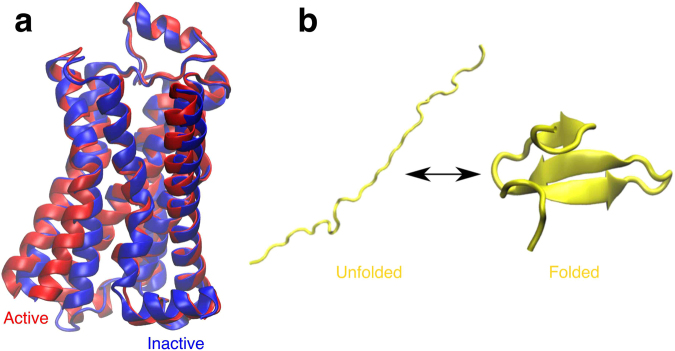
Conformational endpoints in *β*_2_-AR activation and FiP35 WW domain folding. (**a**) Structural alignment of the three-dimensional structures of active (red, PDB ID: 3P0G^52^) and inactive (blue, PDB ID: 2RH1^74^) *β*_2_-AR. (**b**) Three-dimensional structure of the FiP35 WW domain in an arbitrary unfolded state and the folded, native structure.

In recent years, numerous crystal structures have shed light on the inactive states of various GPCRs while limited active state structures have been crystalized^70^. The inherent instability of active state GPCRs without the presence of G proteins makes obtaining active state GPCR crystal structures difficult^52^. Active state structures can potentially be predicted using MD simulation initiated from inactive crystal structures (Fig. 2a), but in practice this approach is computationally expensive due to the long timescales involved in the relevant conformational changes. In order to evaluate the use of directional information encoded in evolutionary couplings for enhancing sampling, we employed different sampling methods with Monte Carlo simulations of *β*_2_-AR using a reference MSM built from 166 *μs* of previously published all-atom molecular dynamics simulations. We calculated first-passage times from the inactive state to the active state for the three sampling methods of interest to determine whether evolutionary couplings-guided adaptive sampling can reduce the time necessary to observe a transition from the inactive to the active state.

**Figure 3:**
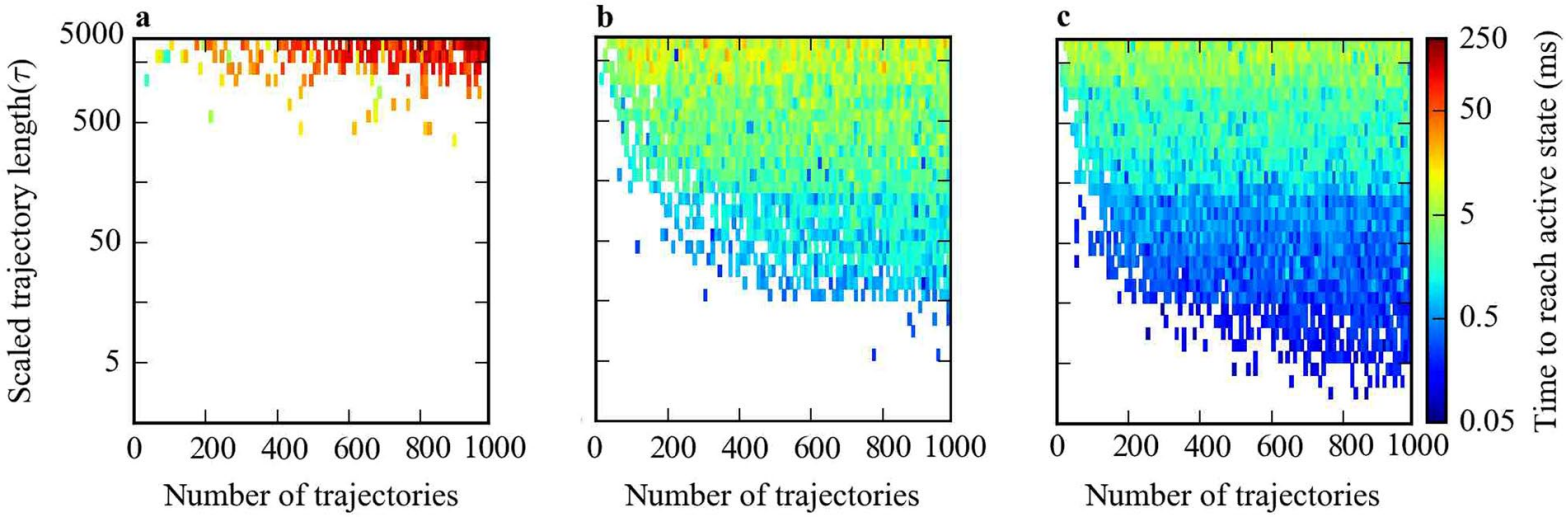
Time to reach the active state of *β*_2_-AR from an inactive state for different sampling methods. (**a**) Traditional long MD simulations, (**b**) random adaptive sampling, and (**c**) evolutionary couplings-guided adaptive sampling. Scaled trajectory length is the length of each trajectory in a specific sampling scheme in terms of the model lag time (*τ*) and number of trajectories is the total number of trajectories run for each specific scheme, given by the product of the number of parallel trajectories and the number of sampling rounds.

Figure 3a shows the required time for multiple parallel trajectories without adaptive sampling to reach the active state. One can immediately see that reaching the active state from inactive is computationally expensive using traditional long simulations. No simulation sets with trajectories shorter than 1000 *τ* (corresponding to 50 *μ*s of MD simulation) ever reached the active state even when 1000 trajectories were run in parallel. Only for trajectories longer than 1000 *τ* was the active state conformation ever reached. An estimate of the first-passage time to the active state in this region of the plot is ~4000 *τ* (200 *μ*s), and as ~800 parallel trajectories were running the total simulation time required to reach the active state was approximately 160 ms. This suggests that reaching the active state of *β*_2_-AR and related GPCRs from the inactive state using traditional molecular dynamics simulations is impractical by current standards.

The computational cost of discovering the active state through kinetic Monte Carlo was reduced when using adaptive sampling. By using the random adaptive sampling method, we saw a decrease in the time to reach the active state by almost two orders of magnitude (Fig. 3b) as compared with the serial trajectory method in addition to discovery of the active state for a wide range of seeding parameters where the active state was never reached using long trajectories. For example, sets of trajectories with lengths of 10 *τ* to 1000 *τ*, which would almost never reach the active state with traditional MD simulation, reached the active state in the comparatively reasonable (though still largely intractable) time of 2 ms using random adaptive sampling.

Though random adaptive sampling successfully discovers the active state, evolutionary couplings guided sampling was considerably more powerful in our simulations. As mentioned previously, distances between evolutionary coupled residues can be considered natural reaction coordinates, which can in principle efficiently guide adaptive sampling in order to discover a biologically functional conformational state. Our results were consistent with this view, as adaptive sampling guided with evolutionary couplings reached the active state an order of magnitude faster than random adaptive sampling and three orders of magnitude faster than traditional MD (Fig. 3c). The active state was discovered with 5 *τ* to 10 *τ* trajectory lengths using evolutionary coupling- guided sampling in a total simulation time of ~0.05 ms, a time far shorter than any observed with traditional MD or random sampling.

The first-passage plots for random and evolutionary couplings-guided adaptive sampling have clear gradients in first-passage time with the trajectory length, where as the length of each trajectory increases the time required for reaching an active state also increases. In order to understand this phenomenon, consider in Fig. 3b the line *x* = 1, representing the system after one round of adaptive sampling (with 10 parallel trajectories). Regardless of trajectory length, there is negligible probability of reaching an active state in the first round of sampling. After the first round, the total passed time for a set of simulations is the number of parallel simulations, times the length of each trajectory and so the total passed time after the first round has a higher value for simulations with longer trajectories. Naturally, if trajectories of all lengths reach the active state in the second round, simulations with longer trajectories will take more total simulation time than simulations with shorter trajectories. The observed rainbow pattern in Fig. 3b,c therefore implies that the number of sampling round plays a more important role in reaching the active state than the length of trajectories.

### Efficient sampling of Fip35 WW domain folding pathways

**Figure 4:**
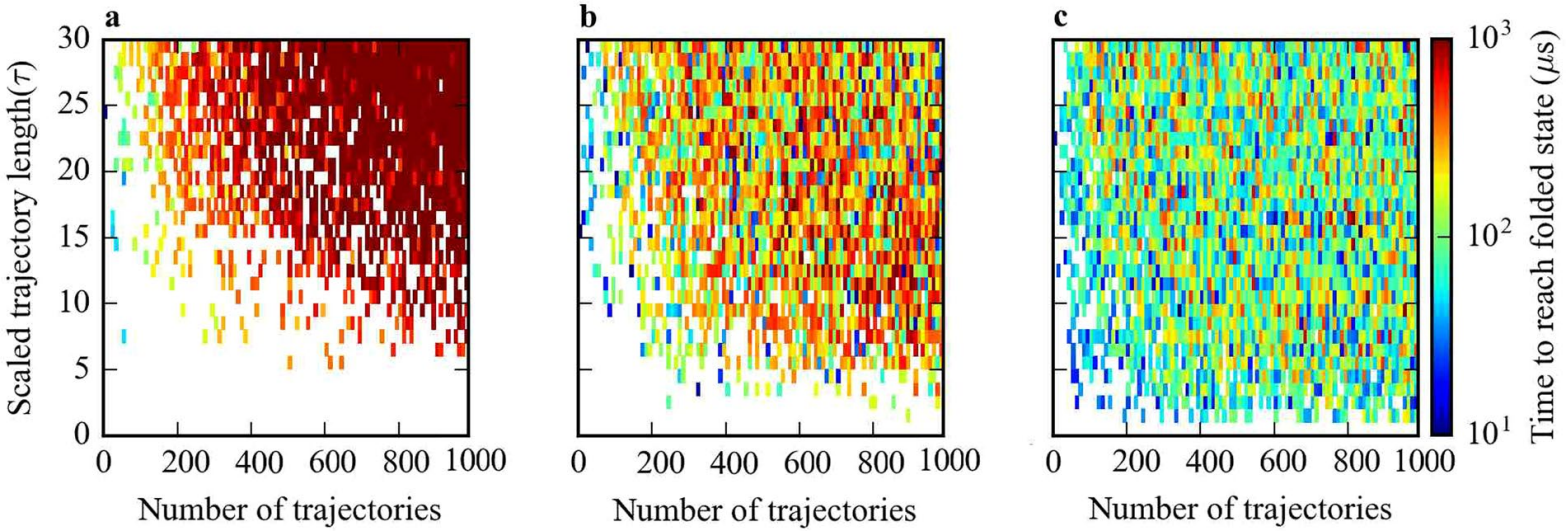
Time to reach the folded state from an arbitrary unfolded state of the FiP35 WW domain for different sampling methods. Shown for sets of simulations using (**a**) traditional long MD simulations, (**b**) random adaptive sampling, and (**c**) evolutionary couplings-guided adaptive sampling. Scaled trajectory length is the length of each trajectory in a specific sampling scheme in terms of the model lag time (*τ*) and number of trajectories is the total number of trajectories run for each specific scheme, given by the product of the number of parallel trajectories and the number of sampling rounds.

Next, we tested ECAS on an MSM built from previously published extensive MD simulation of the FiP35 WW domain folding process^41^ (Fig. 2b) in order to determine the feasibility of using evolutionary couplings to accelerate sampling of folding pathways. As with *β*_2_-AR activation, we found that the time to the folded state from an arbitrarily chosen unfolded state was greatly reduced by using either random or evolutionary couplings-guided adaptive sampling over long serial trajectories with equivalent amounts of simulation time (Fig. 4). Furthermore, there is a clear decrease in the time taken to the folded state for evolutionary couplings-guided adaptive sampling over random adaptive sampling in the regime of short trajectories (Fig. 4b,c), suggesting that evolutionary couplings-guided adaptive sampling provides the greatest improvement in folding speed with many rounds of short trajectories, effectively increasing the amount of adaptive seeding for a given total amount of simulation. Interestingly, there seems to be little change in folding time with increased trajectory length for either the random or evolutionary couplings-guided adaptive sampling methods, while longer trajectories appeared to increase the time taken to reach the active state for these two methods in simulations on the *β*_2_-AR MSM. This is possibly due to the shorter timescales involved in WW domain folding as well as differences in topology between the WW domain MSM describing a folding process and the *β*_2_-AR MSM describing folded conformational changes, where topology has been demonstrated in general to have a notable effect on sampling^71^.

The notion that a well-constructed MSM can reflect the dynamics of the atomistic simulations used to construct them very well gives credence to the use of evolutionary couplings in adaptive sampling for protein folding^44^, though it is unclear how this particular method of choosing structures with the lowest distances between evolutionarily coupled residues will affect sampling of alternate folding pathways that do not follow a monotonic gradient in evolutionary coupling distances. Nonetheless, evolutionary couplings could provide information needed to predict protein folding pathways in less time then is often required for folding, potentially allowing for study of systems for which the folding time is otherwise prohibitive^72^.

### Effects of the number of couplings on sampling

In the previous two sections, we have arbitrarily chosen the residue pairs corresponding to the top 800 ranked evolutionary couplings for use in evolutionary couplings-guided adaptive sampling. In order to evaluate the effects of varying the number of residue pairs used in sampling on the efficiency of the method, we ran further kinetic Monte Carlo simulations on the *β*_2_-AR MSM, using the top ranked 50, 400, 800, 1200, and 1600 residue pairs. We find that with the 50 highest-scoring residue pairs the time to the active state is very similar to random adaptive sampling across the trajectory length/simulation round plane (Supplementary Fig. S8a and b). Of the different numbers of residue pairs tested, 400 gave the most consistently low time to the active state (Supplementary Fig. S8c), while higher numbers of pairs gave similar profiles but more commonly failed to reach the active state at all (Supplementary Fig. S8d–f).

We ran a similar set of simulations on the FiP35 WW domain MSM, using 10, 30, 50, 90, 110, and 272 (all residue pairs with evolutionary coupling between them) residue pairs, finding that using even 10 distances between evolutionarily coupled residue pairs drastically improved sampling (Supplementary Fig. S9a and b) over random adaptive sampling. Increasing the number of coupled residue pairs used from 10 had little noticeable effect on sampling (Supplementary Fig. S9c–g), though when all coupled residue pairs were used folding times resembled those from random sampling.

Additionally, we repeated the same procedure for an MSM built from previously published MD simulation of *λ*-repressor folding^73^, using 230, 250, 270, 290, and 310 coupled residue pairs. While there is a clear improvement over random adaptive sampling, we find little difference between the different numbers of residue pairs (Supplementary Fig. S10).

### Effects of multiple sequence alignment size on sampling

As a GPCR, *β*_2_-AR possesses a wealth of available homologous sequences (amounting to an MSA with 46,610 sequences), allowing for robust evolutionary coupling calculations. However, not all proteins have similarly rich homologous sequence information available, and previous studies have demonstrated a direct relationship between the number of sequences in the MSA used for evolutionary coupling calculations and the quality of folds predicted using those couplings^19^. In order to investigate the effects of MSA size on the sampling efficacy of ECAS, we ran kinetic Monte Carlo simulations on the *β*_2_-AR MSM, this time recalculating evolutionary couplings with MSAs randomly truncated to 20%, 40%, 60%, and 80% of the total homologous sequences from the full MSA and looking for changes in the time taken to the active state using distances between 800 evolutionarily coupled residue pairs. Sampling on *β*_2_-AR is highly sensitive to differences in MSA size, where sampling with couplings calculated from 80% or fewer (Supplementary Fig. S11b–e) of the full number of sequences fared marginally better than random adaptive sampling (Supplementary Fig. S11a).

We repeated the same procedure for the FiP35 WW domain folding MSM, using 70 coupled residue pairs chosen according to evolutionary couplings calculated from MSAs with 20%, 40%, 60%, and 80% of the total homologous sequences from the full MSA. Surprisingly, we find little change in sampling performance with coupling quality (Supplementary Fig. S12).

### Evolutionary couplings-guided sampling provides a near-crystal structure pose and association pathways for the MoaD-MoaE complex

**Figure 5:**
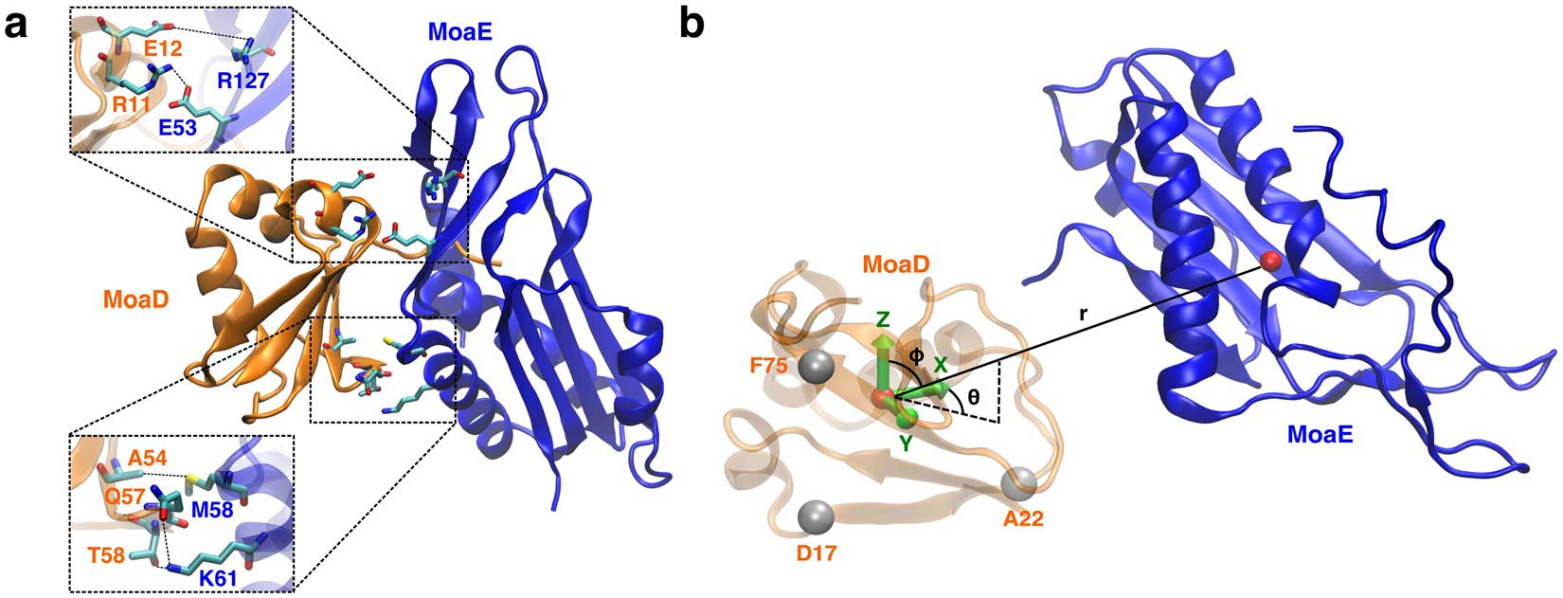
Evolutionary couplings and system representation of the *E. coli* molybdopterin synthase subunits. (**a**) The crystal structure (PDB ID: 1FM0^61^) of MoaD (orange) and MoaE (blue) with the top five scoring evolutionarily coupled pairs displayed. (**b**) The coordinate system used for MSM analysis of MoaD-MoaE association, where a basis set was formed using the *α*-carbons of three MoaD residues and the dynamics of the system were described using the coordinates of the MoaE center of mass vector projected onto the MoaD basis.

**Figure 6:**
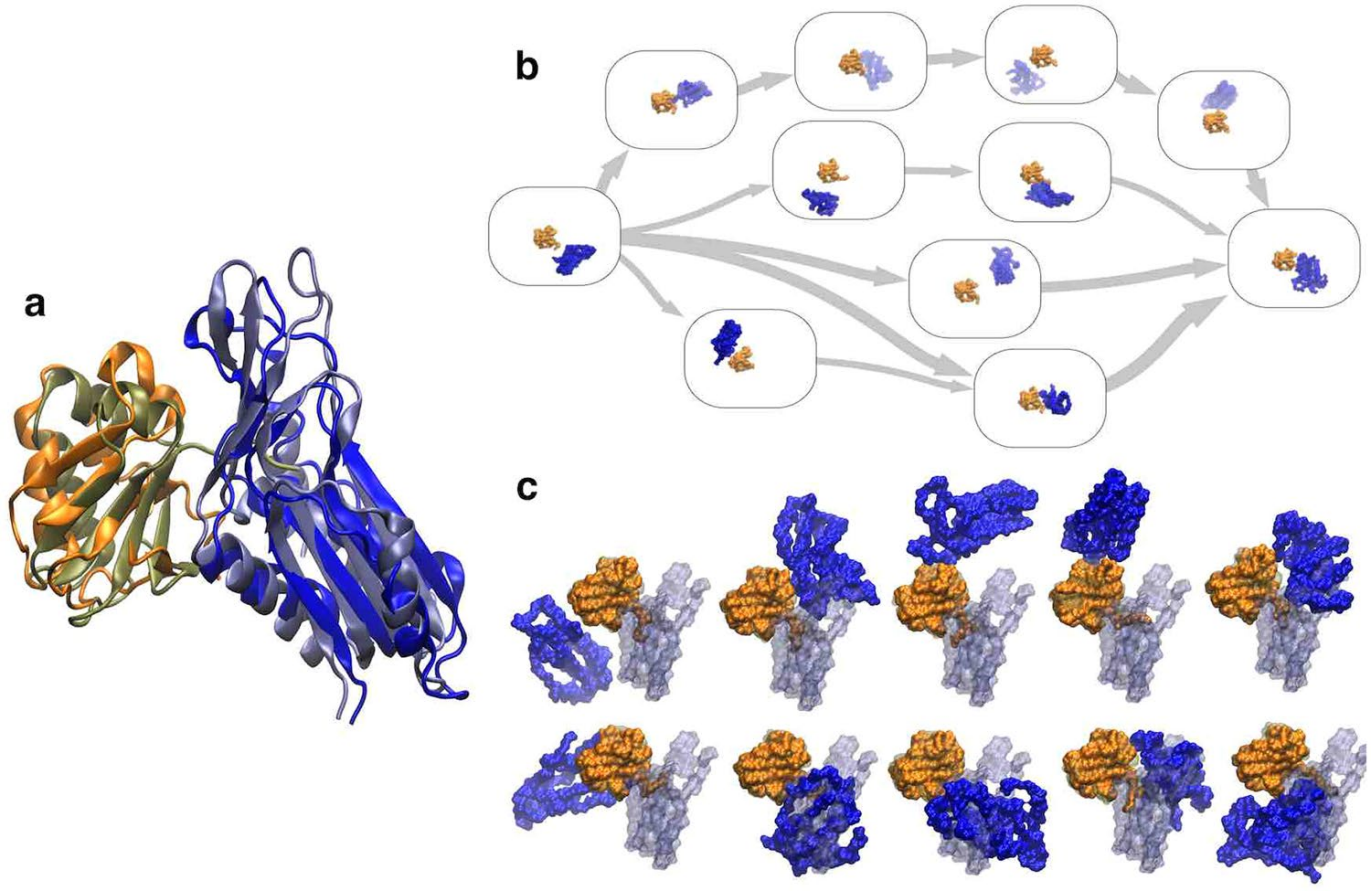
Results from MoaD and MoaE association simulations. (**a**) Superposition of the predicted active structure predicted by selecting the frame with the lowest sum of distances between evolutionary coupled residues with the crystal structure (PDB ID: 1FM0^61^). The backbone RMSD between the two structures was 6.4 Å. (**b**) The five most probable pathways between the state containing the initial separated structure and the state containing the near-crystal structure conformation determined using TPT in the MSMExplorer application^69^. Randomly chosen structures from each state are displayed in the state boxes. (**c**) Randomly chosen structures from the ten states with the highest stationary probabilities calculated from the MSM transition matrix. The crystal structure is superimposed over each structure in a transparent representation for comparison.

Multiple rounds of evolutionary coupling-guided adaptive sampling with all-atom MD simulation of MoaD-MoaE (Fig. 5) association were performed for an aggregate simulation time of 55 *μ*s. By the third round of adaptive sampling, the structure with the minimal sum of coupled residue distances (56.4 Å) (residue pairs shown in Fig. 5a) and the structure with the lowest backbone atom RMSD from the crystal structure (5.8 Å) (Fig. 6a) were realized, where the structure with the minimal sum of coupled distances had a near-minimal backbone atom RMSD from the crystal structure (6.4 Å). Subsequent adaptive sampling rounds did not yield any lower sums of coupled distances or RMSDs to the crystal structure, but contributed to sampling of the ensemble of complex structures and pathways we set out to generate. The cumulative mean sums of distances between the top five evolutionarily coupled residues decreased as a function of adaptive sampling round, from a mean sum of 484.9 Å in the first round to a cumulative mean sum of 398.5 Å by the final round. This result is to be expected from a combination of adaptive sampling along the inter-monomeric distances and removing frames where the distance between the MoaD and MoaE centers of mass was larger than 50 Å from data to be clustered in the adaptive sampling procedure, and could easily be a result of the fact that any vector reaching from one monomer to the other will not be orthogonal to the vector between the two centers of mass of the monomers. We also simulated the MoaD-MoaE complex starting from the crystal structure for 427 ns and found that the RMSD with respect to the crystal structure was distributed with a mean of 3.01 Å and a variance of 0.26 Å^2^, demonstrating that RMSD to the crystal structure as a metric for similarity to the native state does not account for the dynamic nature of the dominant bound state (see Supplementary Fig. S6).

The correspondence between the structure with the minimum sum of coupled residue distances and the near-minimum RMSD structure to some degree demonstrates the power of using evolutionary couplings to predict native protein complexes for this particular protein^22^. However, the advantage of using MD simulations to predict complex structures is that the dynamics inherent to actual protein behavior is captured, revealing pathways to the native structure and additional metastable complex structures. In order to characterize this ensemble of pathways and complexes in a human-understandable manner, we constructed an MSM based on Cartesian coordinates of the MoaE center of mass on a basis set defined by the topology of MoaD. It is important to note that this particular model does not necessarily give converged kinetic or thermodynamic information but rather displays the multiple pathways that the two monomers can take to reach the crystal structure bound state from unbound structures. Though the crystal structure-like state had a relatively high stationary probability, other bound states had comparable stabilities and could represent intermediates in the activation pathway of *E. coli* molybdopterin synthase (Fig. 6c). As insertion of the MoaD C-terminal tail into the active site of MoaE is necessary for MPT synthase catalysis so that the multiplicity of the active state is very low, the near-crystal structure conformation also likely represents an intermediate state in activation^61^. Finally, we used transition path theory to identify the five highest flux pathways from the state with a centroid closest to the initial structure to the state with a centroid closest to the crystal structure (Fig. 6c).

## Discussion

In this study, we have demonstrated the utility of using distances between evolutionarily coupled residues as reaction coordinates for adaptive sampling in molecular simulation, extending the use of evolutionary couplings from generation of static structures to accelerating sampling of folding, activation, and association pathways. True *de novo* predictions of new structures on conformational change or folding pathways are beyond the scope of this study as further extensive molecular dynamics simulations would be required. Although we have sampled association pathways for MoaD-MoaE dimerization in full atomistic detail and recovered a near-crystal structure conformation using ECAS, it is difficult to assess the relevance of any uncovered intermediate structures as 55 *μ*s is almost certainly not enough sampling to calculate converged thermodynamic properties from unbiased simulation. Future studies using ECAS, forthcoming from our group, are needed to demonstrate its use in making novel structural predictions.

The use of distances between evolutionarily coupled residues considerably accelerated sampling on MSMs built from extensive atomistic simulation of the *β*_2_-AR and of the FiP35 WW domain, showing that at least on these two models of protein dynamics, seeding simulations by the distances between coupled residues in adaptive sampling is far more efficient than random seeding choices. Despite the relatively small difference between the active and inactive states (1.524 Å backbone RMSD, PDBIDs: 3P0G^52^ and 2RH1^74^ respectively), the activation time of agonist-bound *β*_2_-AR without a G-protein is believed to be greater than 40 ms^75,76^ while activation is difficult to capture with unbiased simulations^38^. Our simulations suggest that one could capture the activation process of *β*_2_-AR in full atomistic detail in a much shorter time of 50–300 *μ*s. However, we also find that our method is expected to characterize WW domain folding in ~10 *μ*s, which is close to the experimental folding time^77^. While there was no direct evidence that the same acceleration of sampling can occur when this method is applied to protein-protein association, a simple diffusion timescale calculation assuming a fixed oriented encounter rate between proteins would show that the association timescales are longer than the 55 *μ*s simulations performed in this study. With this limited simulation time, we were able to demonstrate that adaptive sampling on evolutionarily coupled residue distances allowed us to identify dominant bound states and binding pathways of the molybdopterin synthase subunits MoaD and MoaE.

In terms of our specific approach for atomistic protein-protein association, a significant amount of computing time was employed in simulating the two monomers freely diffusing in solvent with a considerable distance between them. Our method of taking starting structures for each round of sampling from within a 50 Å radius of the MoaD center of mass partially dealt with this issue. However, it may prove advantageous in future studies to introduce a half-harmonic potential limiting the distance between monomers in order to improve simulation efficiency. Finally, simulations of multiple proteins in general are limited by the accuracy of force fields when treating inter-protein interactions^78,79^.

Knowledge of metastable intermediate states and pathways of activation in dimer formation is essential for understanding protein-protein association in the same way it is for understanding the activation process of a single protein, where relative rigid body translations and rotations together with coupled internal conformational changes of the interacting proteins take the place of the comparatively simple internal conformational changes of a single protein. This knowledge allows for understanding of the mechanisms of activation, including estimation of the kinetics and thermodynamics of the process, and provides insight into how perturbations, such as mutations or binding of small molecules, could affect association. In using evolutionary couplings to guide dimerization of MoaD and MoaE, we were able to both predict the experimentally determined active-like state with moderate accuracy and determine states and pathways likely involved in the association process. This procedure, or some variation, can be used to characterize the association process of two or more known interacting proteins with available individual structures but no complex structure. Adaptive sampling ensures that the degrees of freedom that theoretically define a functional bound state are well sampled but avoids the influence on sampling of other degrees of freedom that restrained simulations introduce, and therefore is an extension to methods of creating protein ensembles from evolutionary couplings from previously published work.

Clearly, the utility of our approach is limited both by the accuracy of evolutionary coupling calculations themselves and in the assumption that evolutionarily coupled residues are likely in spatial proximity in native protein structures. The accuracy of the both the mean field approximation and pseudolikelihood maximization approaches have been validated through their use in accurately predicting folds of numerous proteins^19,20,23,80^, where the assumption of spatial proximity of coupled residues is necessarily used in prediction. There has also been great success in prediction of heteromeric protein complex structures using evolutionary couplings, although these predictions have thus far been restricted to bacterial proteomes^21,22^.

Despite these successes, evolutionary coupling analysis does not produce accurate results for all proteins due to issues with availability of homologous amino acid sequences, limiting the effectiveness of our method to proteins which can be properly analyzed for evolutionary couplings^81^. This issue would be resolved with further availability of homologous sequences, a reasonable prospect for many proteins given the rapid pace at which new sequences are being generated^82^. Still, this limitation of the method is highlighted by the notable drop in performance in time taken to reach the active state of *β*_2_-AR using an MSA with 80% or less of the total number of homologous sequences to calculate evolutionary couplings (Supplementary Fig. S11), especially given that many proteins of interest will likely have fewer homologous sequences available. Interestingly, the quality of couplings appear to have little to no effect on sampling of the WW domain (Supplementary Fig. S12). This could be because evolutionary couplings only guide choice of residue pairs in our method so that for short peptides with few total choices of residue pairs, the probability of choosing important distances for sampling without knowledge of evolutionary couplings is high enough to improve sampling noticeably.

It is unclear how evolutionary coupling quality will effect other systems. However, although calculating couplings with smaller MSAs decreased speedup in sampling, schemes using these poorer quality couplings fared no worse than random adaptive sampling. ECAS using low quality evolutionary couplings could in general give similar results to random adaptive sampling since these couplings will have little relation to residue coevolution and the choices of residue pairs will be effectively random, though we still see improvements in WW domain sampling. Since random adaptive sampling can provide notable acceleration of sampling over non-adaptive sampling, we believe that ECAS in the worst case scenario with entirely randomly chosen couplings will similarly be more efficient than non-adaptive sampling.

It is also unclear how many coupled residue pairs should be chosen for sampling, although a weak trend arose from kinetic Monte Carlo ECAS using different numbers of residue pairs. In *β*_2_-AR, both too few (50) and too many (>800) residue pairs give worse sampling performance than 400 residue pairs (Supplementary Fig. S8). Too many coupled residue pairs (272) gives comparable sampling performance to random adaptive sampling in the WW domain, though for <110 there appears to be little difference in sampling (Supplementary Fig. S9). The number of residue pairs used appears to have no effect on *λ*-repressor sampling, at least in the range tested (Supplementary Fig. S10). Additionally, there could be a relationship between the size of the MSA used to calculate couplings and the number of coupled residue pairs for ECAS, which could be the focus of future work. It is difficult to make general conclusions from these three cases alone, but it appears that there could be an optimal number of coupled residue pairs somewhere between the minimum and maximum total number, which varies from system to system.

For the best performance of ECAS, the number of coupled residue pairs chosen should be near the optimum. As simulation data is required to directly find the optimal number of residue pairs, we propose two alternate approaches to estimate this parameter. First, one could develop a heuristic by finding the optimal number of residue pairs for a wide range of systems and finding a function that maps system characteristics known *a priori* to optimal residue pair number. Whether such a simple relationship actually exists remains to be determined, and could be the aim of future work. Second, one could use an adaptive approach, where the initial number of residue pairs is chosen arbitrarily and adjusted to be optimal on cumulative sampling. Again, such an approach remains to be developed and tested.

Given that many evolutionarily coupled residues are involved in folding, another concern is that biasing sampling on distances between evolutionarily coupled residues that form non-dynamic contacts in the native state could lead to undesired unfolding of the protein. This issue is largely avoided with adaptive sampling, which exploits fluctuations in reaction coordinates to drive sampling, in that contacts involved in folding will be less likely to have large amplitude fluctuations and will likely have a larger energetic barrier in separation than other residues. However, this would create significant issues if these distances had a potential applied to them, as it is not necessarily evident which evolutionarily coupled residues are involved in folding and which are not.

In future studies, evolutionarily coupled residue distances could be of great use as choices for reaction coordinates for temperature accelerated molecular dynamics or umbrella sampling in systems where little *a priori* knowledge is available and where computing time is limited, although as mentioned before some care will have to be taken to avoid using coupled residues intrinsic to the overall fold of the protein of interest when more invasive methods are used. Overall, improvements in evolutionary coupling analysis methods have proven to be extremely useful in computational biophysics, as determining which conformational states are active or biologically competent and which are not without strong prior information from experiments has previously been difficult.

## Electronic supplementary material

Supplementary Information File
