# Publisher Correction: Enhanced unbiased sampling of protein dynamics using evolutionary coupling information

**DOI:** 10.1038/s41598-017-16214-7

**Published:** 2017-12-19

**Authors:** Zahra Shamsi, Alexander S. Moffett, Diwakar Shukla

**Affiliations:** 1 Department of Chemical and Biomolecular Engineering, University of Illinois, Urbana IL, 61801 USA; 2 Center for Biophysics and Quantitative Biology, University of Illinois, Urbana IL, 61801 USA; 3 Department of Plant Biology, University of Illinois, Urbana IL, 61801 USA; 4 National Center for Supercomputing Applications, University of Illinois, Urbana IL, 61801 USA

*Scientific Reports* **7**:12700; doi:10.1038/s41598-017-12874-7; Article published online 05 October 2017

The original HTML version of this Article contained an error in the title of Figure 1 where the following text was inadvertently inserted:

“comment = “We apologize for this mistake, our final submission PDF had the correct order of images, but we did not fix the order of images in the individual submission process. To be clear, the captions in this proof are in the correct order. However, the actual images are in the wrong order. The order of the images alone should be changed as follows:- The image labeled in the proof as Figure 3 should be Figure 1- The image labeled in the proof as Figure 1 should be Figure 2- The image labeled in the proof as Figure 4 should be Figure 3- The image labeled in the proof as Figure 5 should be Figure 4- The image labeled in the proof as Figure 2 should be Figure 5- Figure 6 has the correct image.”

This error has now been corrected in the HTML version of the Article; the PDF version was correct from the time of publication.

