# Supplementary material for: Enhanced unbiased sampling of protein dynamics using evolutionary coupling information
Source: Sci Rep. 2017 Oct 5;7:12700. doi: 10.1038/s41598-017-12874-7 (PMC5629199; doi:10.1038/s41598-017-12874-7)
Supplement: Supplementary file 1 — Supplementary Information File [file 41598_2017_12874_MOESM1_ESM.pdf]

Supplementary Information for: Enhanced unbiased sampling of protein dynamics using evolutionary coupling information

Zahra Shamsi[1,+], Alexander S. Moffett[2,+], and Diwakar Shukla[1,2,3,4,*]

[1]Department of Chemical and Biomolecular Engineering, University of Illinois, Urbana, IL, 61801, USA
[2]Center for Biophysics and Quantitative Biology, University of Illinois, Urbana, IL, 61801, USA
[3]Department of Plant Biology, University of Illinois, Urbana, IL, 61801, USA
[4]National Center for Supercomputing Applications, University of Illinois, Urbana, IL, 61801, USA
[*]diwakar.shukla@shuklagroup.org
[+]These authors contributed equally to this work

Supplementary Methods

Brownian particle simulations

The external potential on all simulated two-dimensional brownian particles was chosen to be a sum of n two-dimensional Gaussians of the form:

$$V(x,y) = \sum_{i=1}^{n} a_i \exp[b_i(x-d_i)^2 + c_i(y-e_i)^2] \tag{1}$$

where the parameters used in simulations are listed in Supplementary Table S1. Additionally, a half-cubic potential was added to constrain the particle to the rugged portion of the potential energy landscape as follows:

$$V_{constraint}(x,y) = \sum_{i=1}^{2} v_i(x) + \sum_{j=1}^{2} v_j(y)$$

$$v_i(x) = \begin{cases} \frac{k_{x,i}}{3}(x+x_i)^3, & \text{if } |x| > |x_i| \text{ and } \mathrm{sgn}(x) \neq \mathrm{sgn}(x_i) \\ 0, & \text{otherwise} \end{cases} \tag{2}$$

$$v_j(y) = \begin{cases} \frac{k_{y,j}}{3}(y+y_j)^3, & \text{if } |y| > |y_i| \text{ and } \mathrm{sgn}(y) \neq \mathrm{sgn}(y_j) \\ 0, & \text{otherwise} \end{cases}$$

where parameters used in simulations are listed in Supplementary Table S2.

MoaD-MoaE MSM construction

Trajectories from simulation of MoaD-MoaE dimerization were featurized by their centers of mass using the MDTraj Python package.[1] For each frame, the MoaD center of mass vector was subtracted from the MoaE center of mass vector and a set of normalized basis vectors were defined from MoaD atom coordinates as follows:

$$\hat{\mathbf{z}}(n) = \frac{\mathbf{v}_{F75,C\alpha}(n) - \mathbf{v}_{D17,C\alpha}(n)}{|\mathbf{v}_{F75,C\alpha}(n) - \mathbf{v}_{D17,C\alpha}(n)|} \tag{4}$$

$$\hat{\mathbf{x}}(n) = \frac{(\mathbf{v}_{A22,C\alpha}(n) - \mathbf{v}_{D17,C\alpha}(n)) - [(\mathbf{v}_{A22,C\alpha}(n) - \mathbf{v}_{D17,C\alpha}(n)) \cdot \hat{\mathbf{z}}(n)]\hat{\mathbf{z}}(n)}{|(\mathbf{v}_{A22,C\alpha}(n) - \mathbf{v}_{D17,C\alpha}(n)) - [(\mathbf{v}_{A22,C\alpha}(n) - \mathbf{v}_{D17,C\alpha}(n)) \cdot \hat{\mathbf{z}}(n)]\hat{\mathbf{z}}(n)|} \tag{5}$$

$$\hat{\mathbf{y}}(n) = \hat{\mathbf{x}}(n) \times \hat{\mathbf{z}}(n) \tag{6}$$

where $\mathbf{v}_{F75C\alpha}(n)$ is the position of the F75 Cα atom in the nth frame and $\hat{\mathbf{x}}(n)$, $\hat{\mathbf{y}}(n)$, $\hat{\mathbf{z}}(n)$ are orthonormal basis vectors spanning $\mathbb{R}^3$ in the nth frame. MoaD was chosen to define the coordinate system due to the relatively narrow and left-shifted distribution of its RMSD with respect to its crystal structure. The center of mass trajectories were then projected onto the MoaD basis sets and the new trajectories were clustered.

λ-repressor MSM construction and kinetic Monte Carlo

We featurized previously published MD simulations[2] by the distribution of reciprocal interatomic distances (DRID),[3] performed time-lagged independent components analysis (tICA),[4,5] projected the DRID coordinates onto the 4 slowest decorrelating tICs, and created a 300 state MSM with a lag time of 3000 ns. The number of tICs to project onto and the number of MSM states were chosen so as to maximize the variational cross-validation score calculated using Osprey.[6,7] Evolutionary couplings guided adaptive sampling (ECAS) was performed in the same way as for β_2-AR and the FiP35 WW domain. 270 evolutionarily coupled residue pairs (the number of pairs with coupling scores over an arbitrarily chosen cutoff score of 0.012, where the number of couplings was rounded) were used.

Testing the effects of multiple sequence alignment size and number of evolutionarily coupled residue pairs used on ECAS performance

For sampling on β_2-AR and the FiP35 WW domain, we took the original multiple sequence alignments (MSAs) returned by the EVCouplings web server[8] and randomly chose 20%, 40%, 60%, and 80% of the sequences (along with the target sequence) to use for calculation of evolutionary couplings, in order to test the effects of progressively poorer evolutionary coupling quality, and therefore the choice of residue pairs for adaptive sampling, on sampling efficacy. ECAS sampling was done with each resulting set of evolutionarily coupled residue pairs chosen according to the poorer quality couplings. 800 residue pairs were used for β_2-AR and 70 pairs were used for the WW domain. The sizes of MSAs used are listed in Supplementary Table S3.

The number of evolutionarily coupled residue pairs was chosen by taking all residue pairs with coupling scores above an arbitrarily chosen cutoff of 0.01, amounting to 800 pairs for β_2-AR, 70 pairs for the FiP35 WW domain, and 270 pairs for the λ-repressor. We then tested ECAS with a range of the top evolutionarily coupled residue pairs (50, 400, 800, 1200, and 1600 for β_2-AR, 10, 30, 50, 70, 90, 110, and 272 for WW domain, and 230, 250, 270, 290, and 310 for the λ-repressor) to determine the effects of residue pair numbers on sampling performance.

Supplementary Discussion

Biasing of simulations through adaptive sampling

Our method employs adaptive seeding of new trajectories without any restraints; the protein dynamics is entirely produced by the native forcefield of the protein and random forces from the solvent or from use of a Langevin integrator. Adaptive sampling, as used here, periodically resets the simulation to a structure fulfilling some condition after a deterministic, constant waiting time. If we consider κ to be the number of simulation steps between resetting events, in the limit $\kappa \to \infty$ we recover unbiased sampling. Conversely, for $\kappa = 1$ we realize a method reminiscent of Monte Carlo simulation, where a single step of simulation is performed and the starting structure of the next round of sampling is chosen as follows: if the change in the metric guiding sampling has the desired sign, select the newly generated conformation. Otherwise, start the next simulation from the same structure as before. In the $\kappa \sim 1$ regime, adaptive sampling imposes a near monotonically change of the chosen metric towards the desired value, very possibly yielding low probability pathways. However, for $\kappa >> 1$, resetting is far less dominant and should provide a comparatively gentle bias towards the desired values of the adaptive sampling metric. As $\kappa \to \infty$, the disparities in path probabilities estimated from adaptive sampling and unbiased sampling will vanish with sufficient sampling.

As κ is clearly a finite number in any implementation of our method, the probability distribution over protein conformations will be biased by resetting. However, by building an MSM, one can effectively remove the bias introduced by resetting. This is possible because the only MSM parameters estimated from simulations are conditional transition probabilities between states, so artificially starting a simulation from a structure within a state does not directly bias the result. Transitions between states are entirely determined by the stochastic dynamics if the Markov property holds for the MSM, and should therefore be faithful to the unbiased dynamics if the system remains in local equilibrium[9,10]. If the transition probabilities are accurate, then because MSMs are irreducible, reversible Markov chains there will be a unique stationary distribution over the states, which is also accurate. If there is high statistical error in some transition probabilities, the mode of sampling can be changed so that trajectories are seeded from states with high-error transition probabilities in order to refine the model[11]. Additionally, after a path for folding or conformational change is found, further sampling can be initiated from states along the path in order to discover potential alternate routes.

There is no guarantee that the path found by ECAS will be the lowest free energy path of folding or conformational change. However, this is also true for unbiased sampling. By using large κ values and constructing Markov state models to debias simulations, we believe that pathways of folding and conformational change found using ECAS should not significantly differ from unbiased molecular dynamics simulations. Our method is not intended to guarantee optimality; rather, it is intended as a useful heuristic to improve sampling without altering the potential energy function. It is also not intended purely as a structure prediction method; there are far more efficient methods of structure prediction using evolutionary couplings[12].

References

1. McGibbon, R. T. *et al.* Mdtraj: A modern open library for the analysis of molecular dynamics trajectories. *Biophys. J.* **109**, 1528–1532 (2015).
2. Lindorff-Larsen, K., Piana, S., Dror, R. O. & Shaw, D. E. How fast-folding proteins fold. *Science* **334**, 517–520 (2011).
3. Zhou, T. & Caflisch, A. Distribution of reciprocal of interatomic distances: A fast structural metric. *J. Chem. Theory Comput.* **8**, 2930–2937 (2012).
4. Naritomi, Y. & Fuchigami, S. Slow dynamics in protein fluctuations revealed by time-structure based independent component analysis: The case of domain motions. *J. Chem. Phys.* **134**, 065101 (2011).
5. Pérez-Hernández, G., Paul, F., Giorgino, T., De Fabritiis, G. & Noé, F. Identification of slow molecular order parameters for markov model construction. *J. Chem. Phys.* **139**, 015102 (2013).
6. McGibbon, R. T. & Pande, V. S. Variational cross-validation of slow dynamical modes in molecular kinetics. *J. Chem. Phys.* **142**, 03B621_1 (2015).
7. McGibbon, R. T. *et al.* Osprey: Hyperparameter optimization for machine learning. *J. Open Source Softw.* **1** (2016). URL https://doi.org/10.21105%2Fjoss.00034.
8. Sheridan, R. *et al.* EVfold.org: Evolutionary couplings and protein 3D structure prediction. *bioRxiv* doi:10.1101/021022 (2015).
9. Huang, X., Bowman, G. R., Bacallado, S. & Pande, V. S. Rapid equilibrium sampling initiated from nonequilibrium data. *Proc. Natl. Acad. Sci. USA* **106**, 19765–19769 (2009).
10. Nüske, F. *et al.* Markov state models from short non-equilibrium simulations—analysis and correction of estimation bias. *J. Chem. Phys.* **146**, 094104 (2017).
11. Hinrichs, N. S. & Pande, V. S. Calculation of the distribution of eigenvalues and eigenvectors in markovian state models for molecular dynamics. *J. Chem. Phys.* **126**, 244101 (2007).
12. Marks, D. S., Hopf, T. A. & Sander, C. Protein structure prediction from sequence variation. *Nat. Biotechnol.* **30**, 1072–1080 (2012).
13. Cherezov, V. *et al.* High-resolution crystal structure of an engineered human β2-adrenergic g protein–coupled receptor. *Science* **318**, 1258–1265 (2007).
14. Rasmussen, S. G. *et al.* Structure of a nanobody-stabilized active state of the [bgr] 2 adrenoceptor. *Nature* **469**, 175–180 (2011).
15. Rudolph, M. J., Wuebbens, M. M., Rajagopalan, K. V. & Schindelin, H. Crystal structure of molybdopterin synthase and its evolutionary relationship to ubiquitin activation. *Nat. Struct. Biol.* **8**, 42–46 (2001).

Supplementary Table S1. Parameters for each of the 9 Gaussians used for the external potential in brownian dynamics simulations.

i =	1	2	3	4	5	6	7	8	9
a_i	-200	-150	-100	-150	-150	-150	-100	-150	-200
b_i	-3	-3	-3	-3	-3	-3	-3	-3	-3
c_i	-2	-2	-2	-2	-2	-2	-2	-2	-2
d_i	0	1.5	3	0	1.5	3	0	1.5	3
e_i	0	0	0	2	2	2	4	4	4

Supplementary Table S2. Parameters for the half-cubic bounding potentials.

x_1	x_2	y_1	y_2	$k_{x,1}$	$k_{x,2}$	$k_{y,1}$	$k_{y,2}$
1	-4	1.5	-5.5	-200	200	-200	200

Supplementary Table S3. Number of sequences in multiple sequence alignments used for evolutionary coupling calculation.

	β_2-AR	FiP35 WW domain	λ-repressor
20%	9322	1254	–
40%	18644	2508	–
60%	27966	3761	–
80%	37287	5015	–
100%	46610	6268	92335

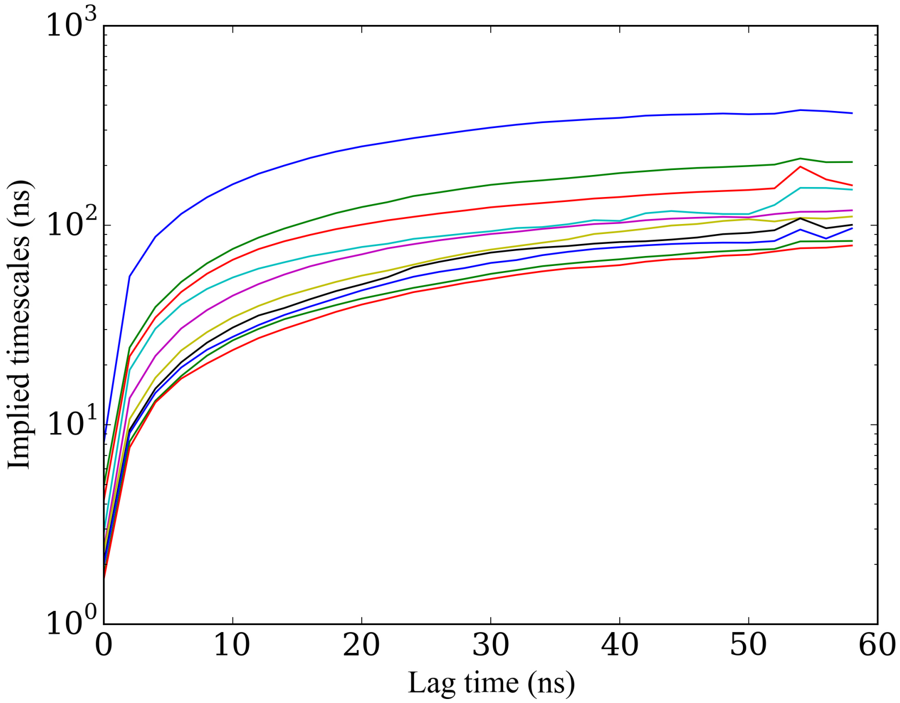

Supplementary Figure S1. Scaling of MoaD-MoaE MSM implied timescales with lag time. A lag time of 40 ns was chosen, as the timescales plateau at this lag time, implying maximization of Markovian behavior of the MSM.

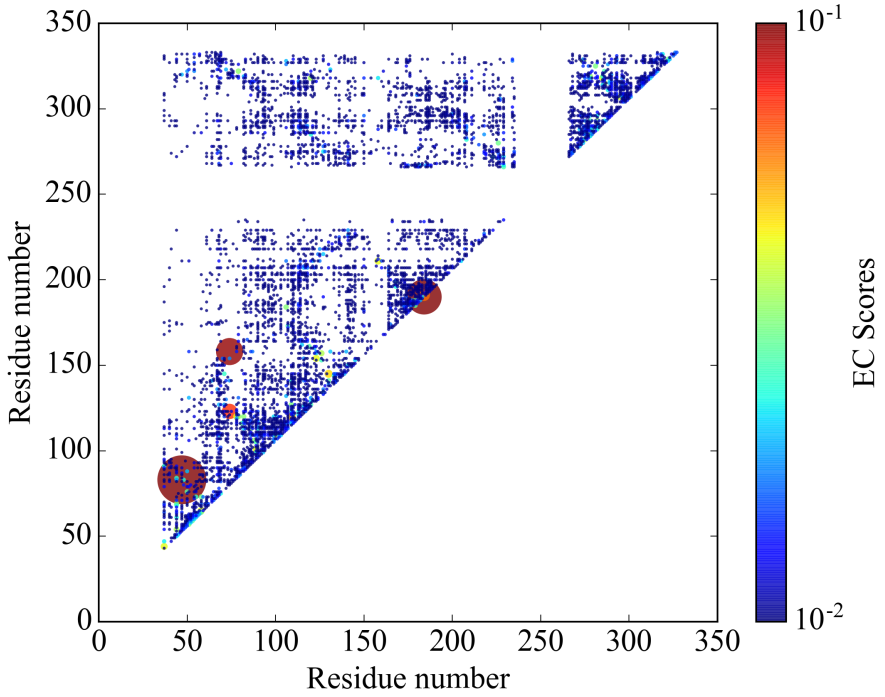

Supplementary Figure S2. Evolutionary coupling scores of 5000 evolutionary couplings with higher evolutionary coupling score values in β_2-AR.

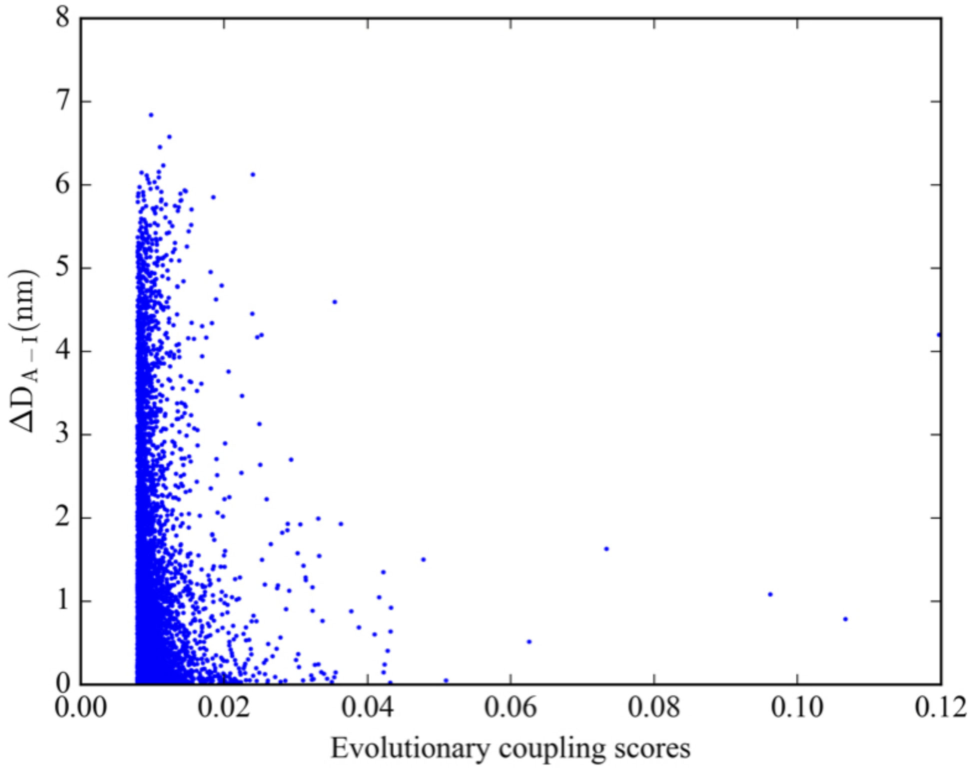

Supplementary Figure S3. Difference in the evolutionary coupling distances between active and inactive crystal structures of β_2-AR. Crystal structures of the active and inactive structures (respectively, PDB IDs: 2RH1[13] and 3SN6[14]) and couplings with evolutionary coupling score values greater than 0.008 were considered in the calculations.

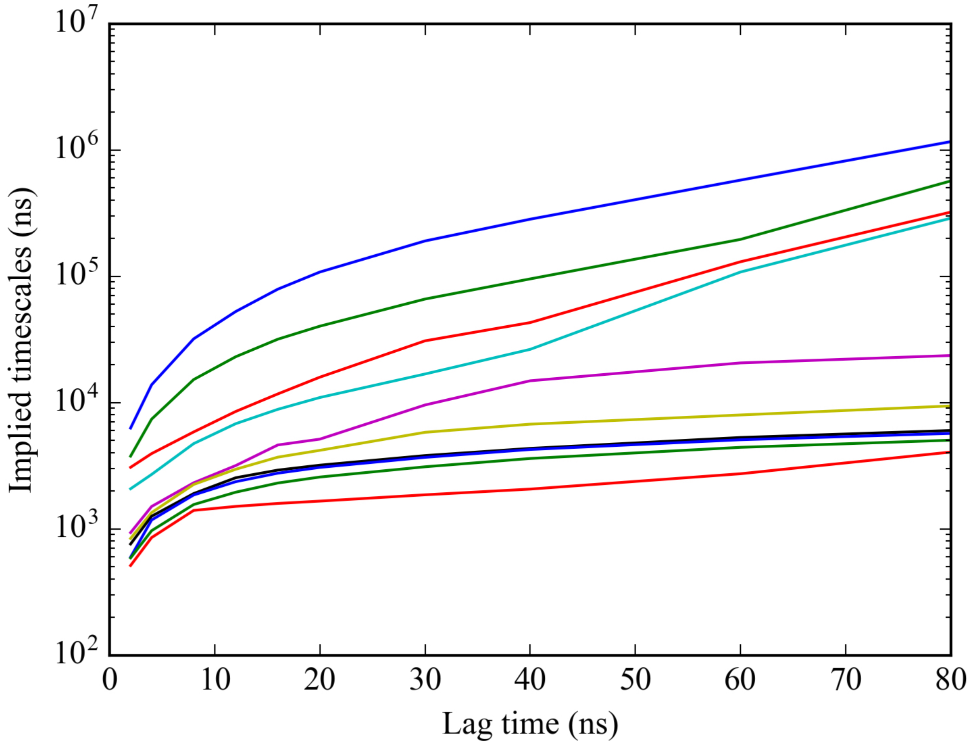

Supplementary Figure S4. Implied timescales for a 1000 state MSM built from β_2-AR simulation data at different lag times. A lag time of 50 ns was chosen for the MSM for this study.

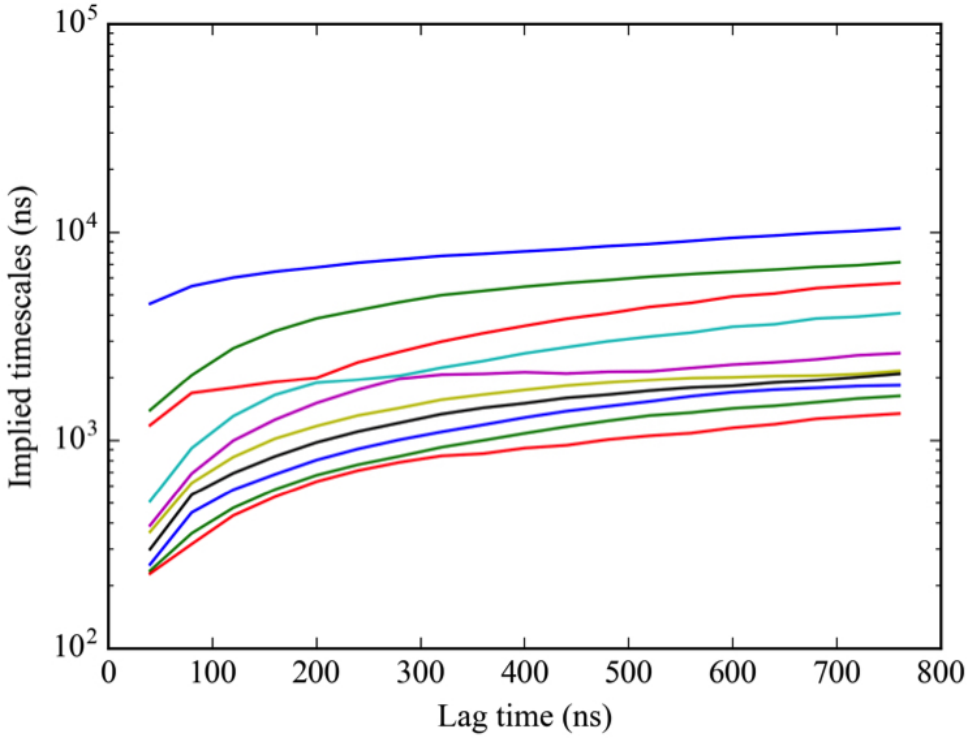

Supplementary Figure S5. Implied timescales for a 2000 state MSM built from WW domain simulation data at different lag times. A lag time of 120 ns was chosen for the MSM for this study.

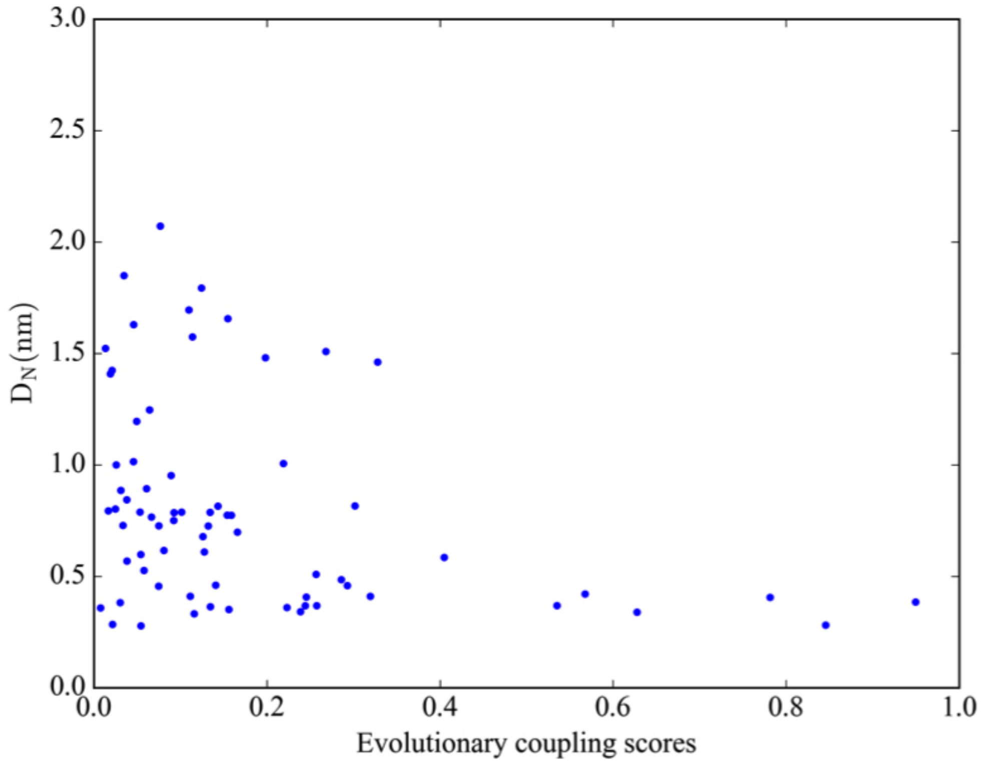

Supplementary Figure S6. Distances between evolutionarily coupled residues in the FiP35 WW domain native folded structure versus evolutionary coupling scores. Couplings with scores above .008 were used in adaptive sampling.

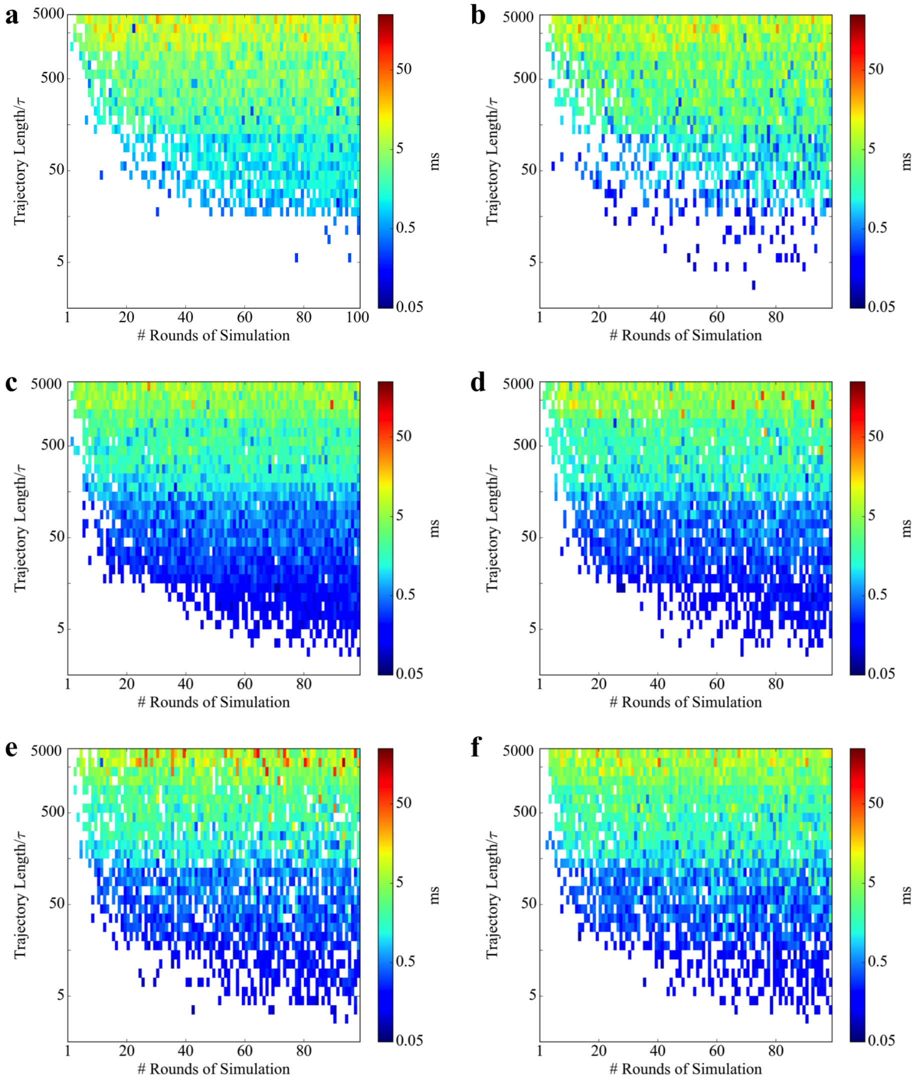

Supplementary Figure S7. Travel time from the inactive to active state of β_2-AR using evolutionary couplings-guided adaptive sampling with different numbers of couplings. Time to the active state in kinetic Monte Carlo simulation on the β_2-AR MSM using the N top-scoring evolutionary couplings, for a) random adaptive sampling and with values of N: b) 50 c) 400 d) 800 e) 1200 and f) 1600. Scaled trajectory length is the length of each trajectory in a specific sampling scheme in terms of the model lag time (τ) and number of trajectories is the total number of trajectories run for each specific scheme, given by the product of the number of parallel trajectories and the number of sampling rounds.

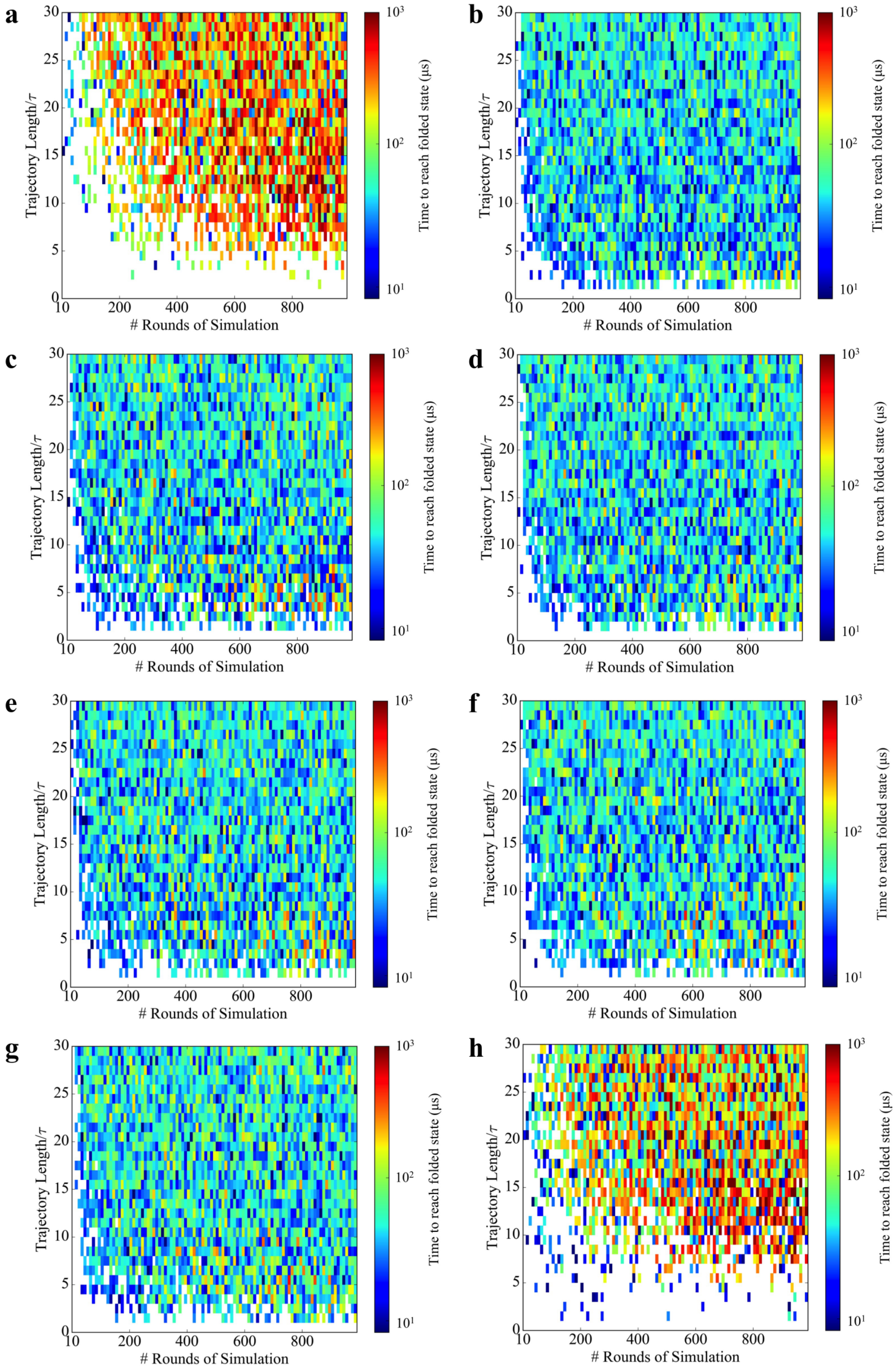

Supplementary Figure S8. Folding time of the FiP35 WW domain using evolutionary couplings-guided adaptive sampling with different numbers of couplings. Folding time in kinetic Monte Carlo simulation on the FiP35 WW domain using the N top-scoring evolutionary couplings, for a) random adaptive sampling and with values of N: b) 10 c) 30 d) 50 e) 70 f) 90 g) 110 and h) 272 (all coupled residue pairs returned by EVCouplings). Scaled trajectory length is the length of each trajectory in a specific sampling scheme in terms of the model lag time (τ) and number of trajectories is the total number of trajectories run for each specific scheme, given by the product of the number of parallel trajectories and the number of sampling rounds.

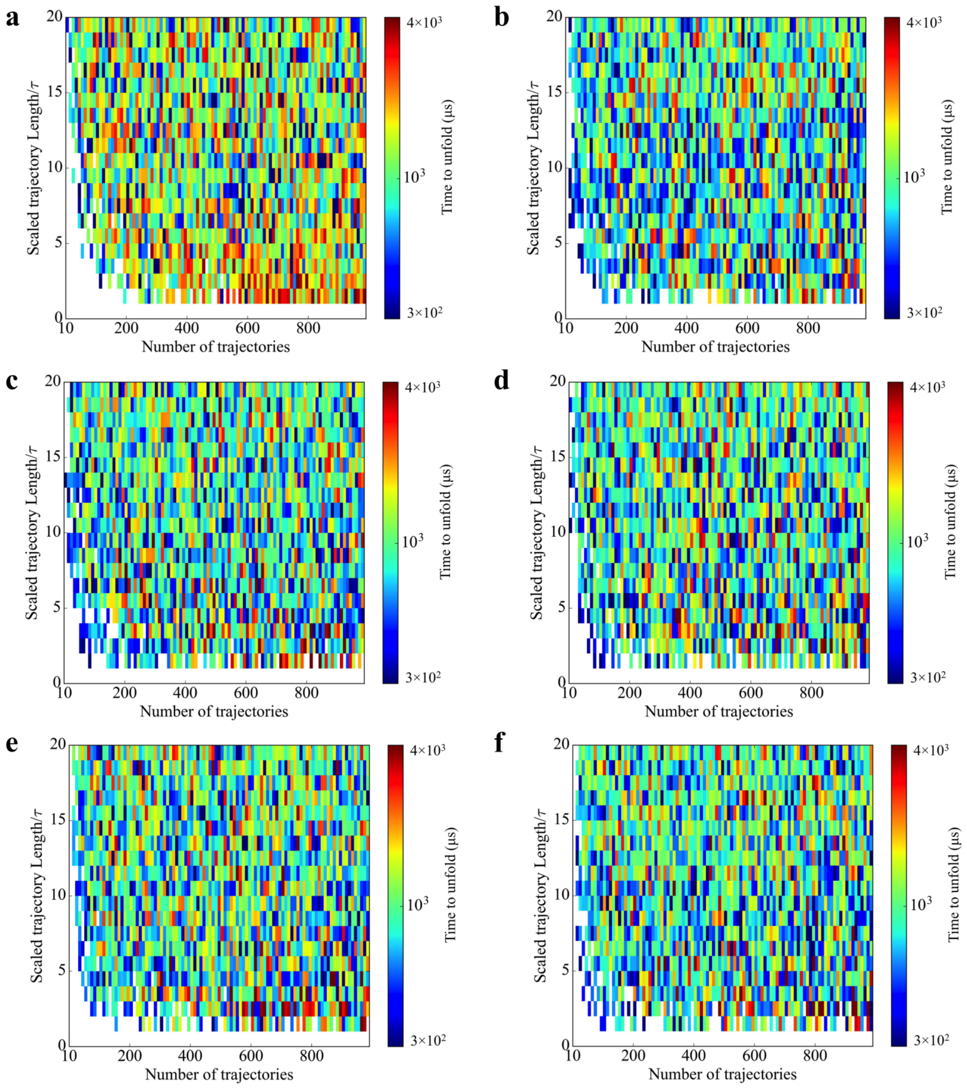

Supplementary Figure S9. Folding time of the λ-repressor using evolutionary couplings-guided adaptive sampling with different numbers of couplings. Folding time in kinetic Monte Carlo simulation on the λ-repressor MSM using the N top-scoring evolutionary couplings, for a) random adaptive sampling and with values of N: b) 230 c) 250 d) 270 e) 290 and f) 310. Scaled trajectory length is the length of each trajectory in a specific sampling scheme in terms of the model lag time (τ) and number of trajectories is the total number of trajectories run for each specific scheme, given by the product of the number of parallel trajectories and the number of sampling rounds.

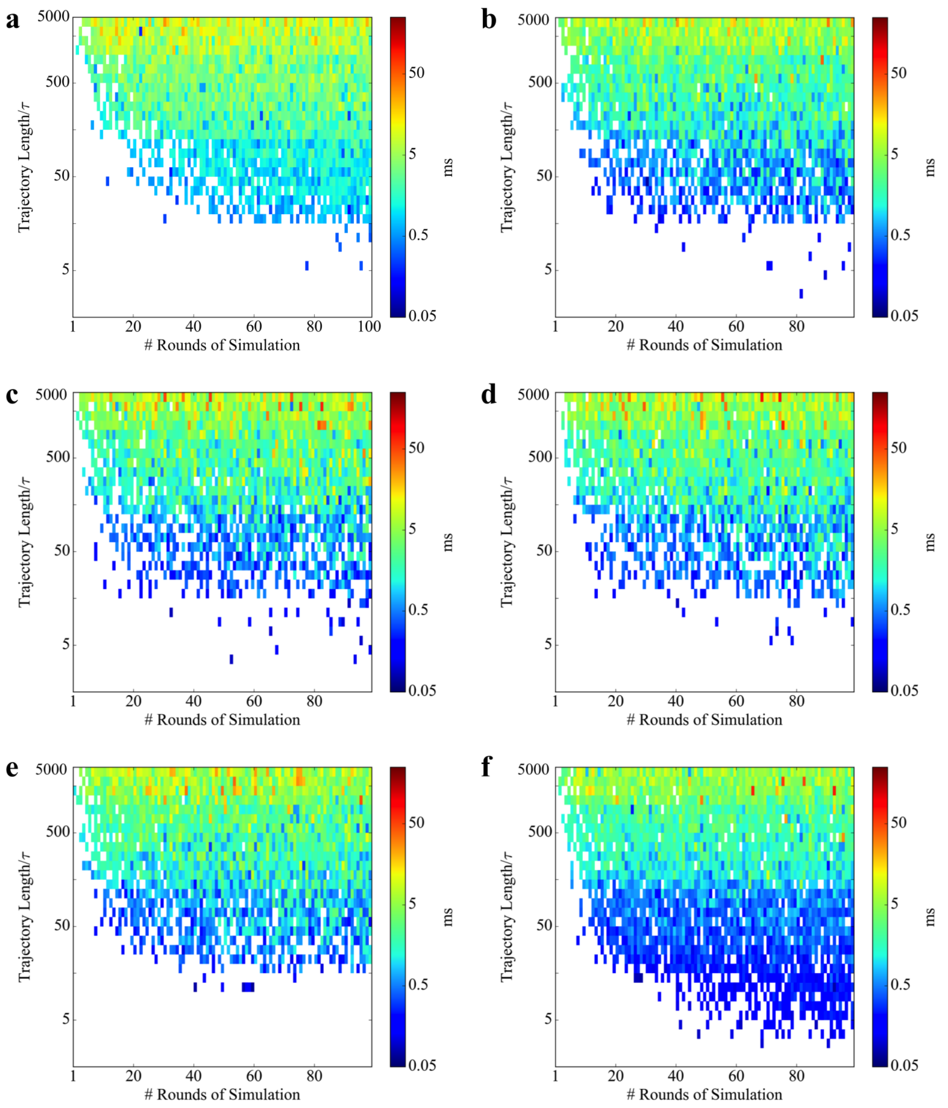

Supplementary Figure S10. Travel time from the inactive to active state of β_2-AR using evolutionary couplings-guided adaptive sampling with couplings calculated from truncated alingments. Time to the active state in kinetic Monte Carlo simulation on the β_2-AR MSM using evolutionary couplings calculated from truncated multiple sequence alignments generated from the full alignment returned by the EVCouplings web server. The truncated alignments were generated by randomly choosing sets of sequences from the full alignment of varying size, given by the percent of the number of sequences in the full alignment: a) 20% b) 40% c) 60% d) 80% and e) 100%. Scaled trajectory length is the length of each trajectory in a specific sampling scheme in terms of the model lag time (τ) and number of trajectories is the total number of trajectories run for each specific scheme, given by the product of the number of parallel trajectories and the number of sampling rounds.

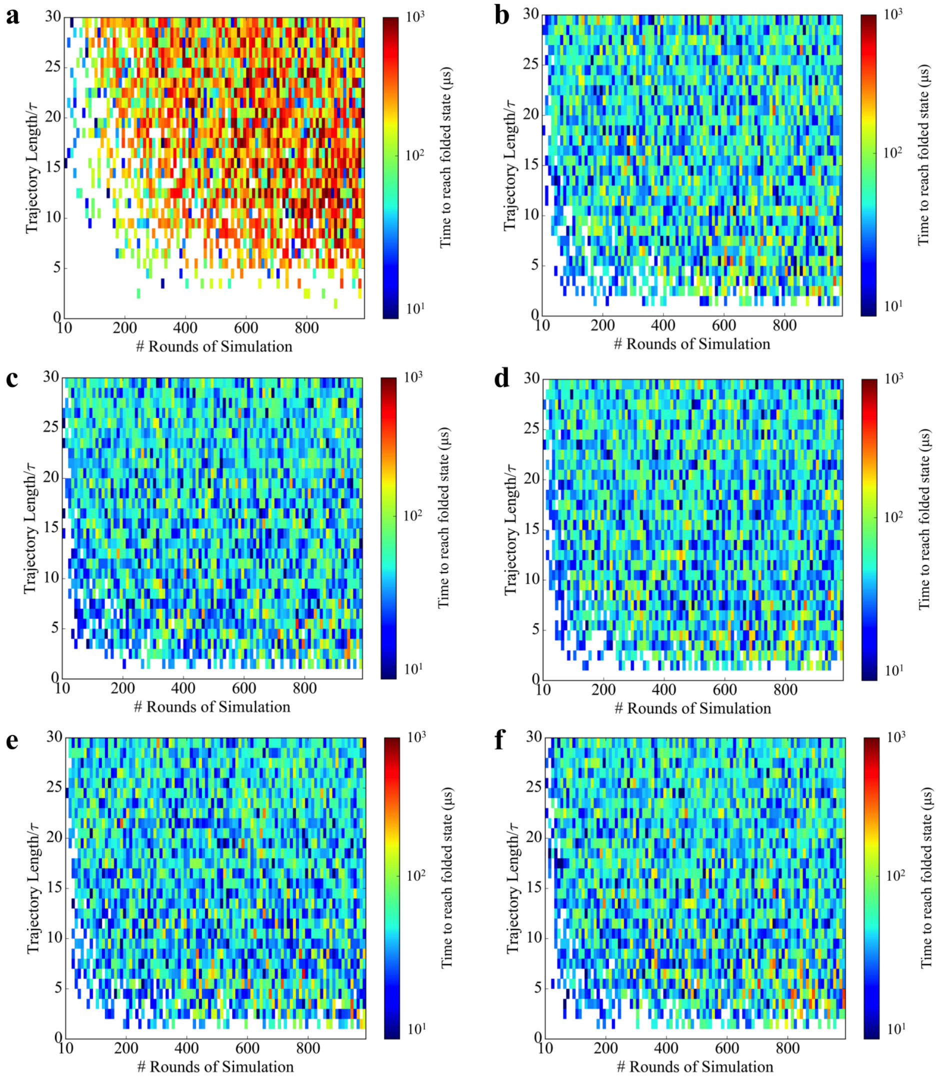

Supplementary Figure S11. Folding time of the FiP35 WW domain using evolutionary couplings-guided adaptive sampling with couplings calculated from truncated alingments. Folding time in kinetic Monte Carlo simulation on the FiP35 WW domain MSM using evolutionary couplings calculated from truncated multiple sequence alignments generated from the full alignment returned by the EVCouplings web server. The truncated alignments were generated by randomly choosing sets of sequences from the full alignment of varying size, given by the percent of the number of sequences in the full alignment: a) 20% b) 40% c) 60% d) 80% and e) 100%. Scaled trajectory length is the length of each trajectory in a specific sampling scheme in terms of the model lag time (τ) and number of trajectories is the total number of trajectories run for each specific scheme, given by the product of the number of parallel trajectories and the number of sampling rounds.

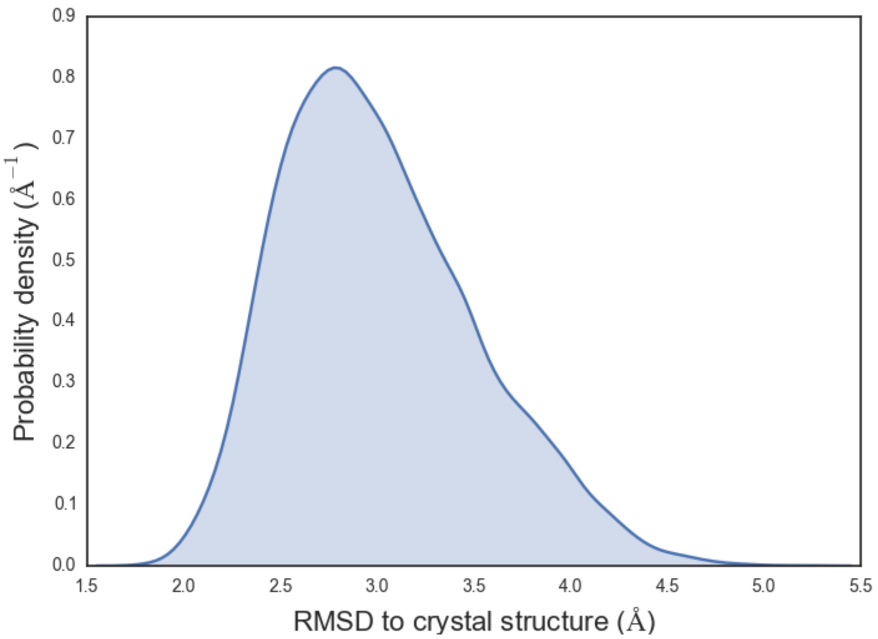

Supplementary Figure S12. Kernel density estimate of the backbone atom RMSD with respect to the crystal structure (PDB ID: 1FM0[15]) from 427 ns of simulation of the MoaD-MoaE dimer starting from the crystal structure.
